# Genetic Variation within Clonal Lineages of *Phytophthora infestans* Revealed through Genotyping-By-Sequencing, and Implications for Late Blight Epidemiology

**DOI:** 10.1371/journal.pone.0165690

**Published:** 2016-11-03

**Authors:** Zachariah R. Hansen, Kathryne L. Everts, William E. Fry, Amanda J. Gevens, Niklaus J. Grünwald, Beth K. Gugino, Dennis A. Johnson, Steven B. Johnson, Howard S. Judelson, Brian J. Knaus, Margaret T. McGrath, Kevin L. Myers, Jean B. Ristaino, Pamela D. Roberts, Gary A. Secor, Christine D. Smart

**Affiliations:** 1 Plant Pathology and Plant-Microbe Biology Section, Cornell University, Geneva, NY, United States of America; 2 Department of Plant Science and Landscape Architecture, University of Maryland, Salisbury, MD, United States of America; 3 Plant Pathology and Plant-Microbe Biology Section, Cornell University, Ithaca, NY, United States of America; 4 Department of Plant Pathology, University of Wisconsin, Madison, WI, United States of America; 5 Horticultural Crops Research Laboratory, United States Department of Agriculture–Agricultural Research Service, Corvallis, OR, United States of America; 6 Department of Plant Pathology and Environmental Microbiology, The Pennsylvania State University, University Park, PA, United States of America; 7 Department of Plant Pathology, Washington State University, Pullman, WA, United States of America; 8 University of Maine Cooperative Extension, Presque Isle, ME, United States of America; 9 Department of Plant Pathology and Microbiology, University of California Riverside, Riverside, CA, United States of America; 10 Plant Pathology and Plant-Microbe Biology Section, Cornell University, Riverhead, NY, United States of America; 11 Department of Plant Pathology, North Carolina State University, Raleigh, NC, United States of America; 12 Department of Plant Pathology, University of Florida, Immokalee, FL, United States of America; 13 Department of Plant Pathology, North Dakota State University, Fargo, ND, United States of America; Agriculture and Agri-Food Canada, CANADA

## Abstract

Genotyping-by-sequencing (GBS) was performed on 257 *Phytophthora infestans* isolates belonging to four clonal lineages to study within-lineage diversity. The four lineages used in the study were US-8 (n = 28), US-11 (n = 27), US-23 (n = 166), and US-24 (n = 36), with isolates originating from 23 of the United States and Ontario, Canada. The majority of isolates were collected between 2010 and 2014 (94%), with the remaining isolates collected from 1994 to 2009, and 2015. Between 3,774 and 5,070 single-nucleotide polymorphisms (SNPs) were identified within each lineage and were used to investigate relationships among individuals. K-means hierarchical clustering revealed three clusters within lineage US-23, with US-23 isolates clustering more by collection year than by geographic origin. K-means hierarchical clustering did not reveal significant clustering within the smaller US-8, US-11, and US-24 data sets. Neighbor-joining (NJ) trees were also constructed for each lineage. All four NJ trees revealed evidence for pathogen dispersal and overwintering within regions, as well as long-distance pathogen transport across regions. In the US-23 NJ tree, grouping by year was more prominent than grouping by region, which indicates the importance of long-distance pathogen transport as a source of initial late blight inoculum. Our results support previous studies that found significant genetic diversity within clonal lineages of *P*. *infestans* and show that GBS offers sufficiently high resolution to detect sub-structuring within clonal populations.

## Introduction

*Phytophthora infestans* is a highly aggressive and destructive pathogen that causes late blight of potato and tomato. Although it has been studied extensively since it was first described in the 19^th^ century [1], it remains one of the most constraining factors in potato and tomato production [2]. A key reason for this is the pathogen’s ability to adapt to disease management practices including host resistance and fungicides [3,4]. Additionally, each late blight lesion is capable of producing hundreds of thousands of wind-dispersed sporangia after as few as five days, causing epidemics to progress very rapidly under favorable conditions [5].

An important aspect of the biology of *P*. *infestans* is the ability to reproduce both sexually and asexually. This allows for genetic recombination via sexual reproduction followed by rapid proliferation of the fittest individuals via asexual reproduction and dispersal via airborne sporangia or movement on infected plant tissue. The resulting clonal lineages, which are comprised of clonal descendants of one unique individual, then dominate a geographic region until a more fit individual displaces them [6,7]. In the United States, where sexual reproduction is not common but has been indirectly observed twice [8,9], novel-genotypes are presumed to emerge through migration [10–12]. These new lineages often display phenotypes that differ from their predecessors in agriculturally-important characteristics such as host preference (tomato vs. potato), ability to overcome host resistance, and fungicide sensitivity [13,14]. Four lineages that have had significant impacts in the United States in recent years are US-8, US-11, US-23, and US-24 [15]. The US-8 and US-11 lineages, which first appeared in 1992 and 1994 [11,16], respectively, have resistance to the commonly used fungicide mefenoxam. The US-23 and US-24 lineages, which first appeared in 2009 [13], are susceptible to mefenoxam [14,16]. Additionally, US-11 and US-23 are both virulent pathogens of potato and tomato, whereas US-8 and US-24 are virulent on potato but are weak pathogens of tomato [13,14,17].

Without sexual reproduction *P*. *infestans* requires living host tissue to survive in the field. In climates where late blight hosts cannot survive the winter, the pathogen can survive in potato tubers, which may be in storage, in cull piles, or left in the ground following harvest [18]. The pathogen’s ability to overwinter in potato tubers and initiate late blight infections the following spring has been known for a long time [19]. Recently, the ability of lineages US-22, US-23, and US-24 to survive extended periods below 0°C in tomato seed was demonstrated under controlled laboratory conditions [20]. However, more work needs to be done to determine whether or not volunteer tomatoes can serve as an overwintering inoculum source in cold climates under field conditions. Long-distance pathogen transport via infected host tissue is also known to occur, as was the case with the HERB-1 mitochondrial lineage responsible for causing the Irish potato famine [21,22], and the subsequent introduction of the US-1 lineage of *P*. *infestans* that was globally distributed in the mid-20th century [22,23]. More recently in 2009, infected tomato seedlings distributed from large retail stores to home gardeners were identified as the cause of a major late blight outbreak in the United States [13,15]. However, the relative importance of regional pathogen overwintering versus long distance transport via infected seed potatoes or tomato transplants with respect to initial inoculum is not well understood.

Historically, genotypic diversity in *P*. *infestans* has been evaluated using allozymes [24], restriction fragment length polymorphisms [11], mitochondrial haplotypes [9,25], and more recently microsatellites [14,26]. The 12 microsatellite markers currently used to genotype isolates of *P*. *infestans* have sufficient resolution to distinguish clonal lineages, and have also been used to investigate diversity within lineages [6,14,27]. Several studies have identified phenotypic variability among asexual *P*. *infestans* progeny [3,28–31]. Genotypic variability among asexual progeny has also been observed, although the number of genetic markers available to investigate such variability (RFLPs and AFLPs) has been relatively low until recently (Abu-El Samen et al., 2003a; reviewed in Goodwin, 1997). Therefore, sub-lineages in natural asexual *P*. *infestans* populations have not been identified.

Genotyping-by-sequencing (GBS) is a relatively new technology which combines reduced representation of the genome with next-generation sequencing for simultaneous marker discovery and individual genotyping [33,34]. This approach, through the identification of thousands of single nucleotide polymorphisms (SNPs), vastly increases the density of genetic markers over previous technologies, such as microsatellites, thereby increasing the resolution available to study population genetics. Since its development GBS has been used to study plant populations [33,35,36] as well as plant pathogen populations [34,37,38].

The overall goal of this project was to utilize GBS to identify SNPs within clonal lineages of *P*. *infestans*, and to use these data to better understand within-lineage genetic diversity. To accomplish this, the neighbor-joining (NJ) method was used to visualize diversity and population structure within each of four dominant clonal lineages. A second objective was to analyze sub-lineage population structure and determine if inferences could be made about late blight epidemiology. Within-lineage groupings were evaluated to gain insight into pathogen overwintering and dispersal patterns.

## Materials and Methods

### Isolates

The majority of isolates used in this study were collected as part of the USAblight project, a national project focused on improving understanding and management of potato and tomato late blight in the USA (http://www.usablight.org). Older isolates (prior to 2011) were obtained from the Cornell University Culture Collection or directly from collaborators. Potato and tomato late blight samples submitted prior to or during the USAblight project were collected by regional cooperators (primarily researchers and cooperative extension educators) and mailed overnight to W. E. Fry at Cornell University for SSR genotyping using markers developed by Lees et al. (2006) (APHIS permit 0579–0054). Following SSR genotyping (clonal lineage assignment) isolates were stored at 16°C in sterile glass vials filled half way with Rye B agar [29] as part of the Cornell University Culture Collection (W. E. Fry). Isolates were selected for this study to maximize temporal and geographic diversity.

Prior to DNA extraction, isolates were removed from storage, transferred onto pea agar [39], and plugs of actively growing mycelia were transferred into pea broth and incubated at 16°C for five to ten days. Mycelia were filtered from pea broth using vacuum filtration and qualitative P8 grade filter paper (Thermo Fisher Scientific, Waltham, MA). Approximately 150 mg wet mycelia per isolate were collected and stored in sterile 2 ml round-bottom tubes at -20°C until DNA was extracted.

**Fig 1:**
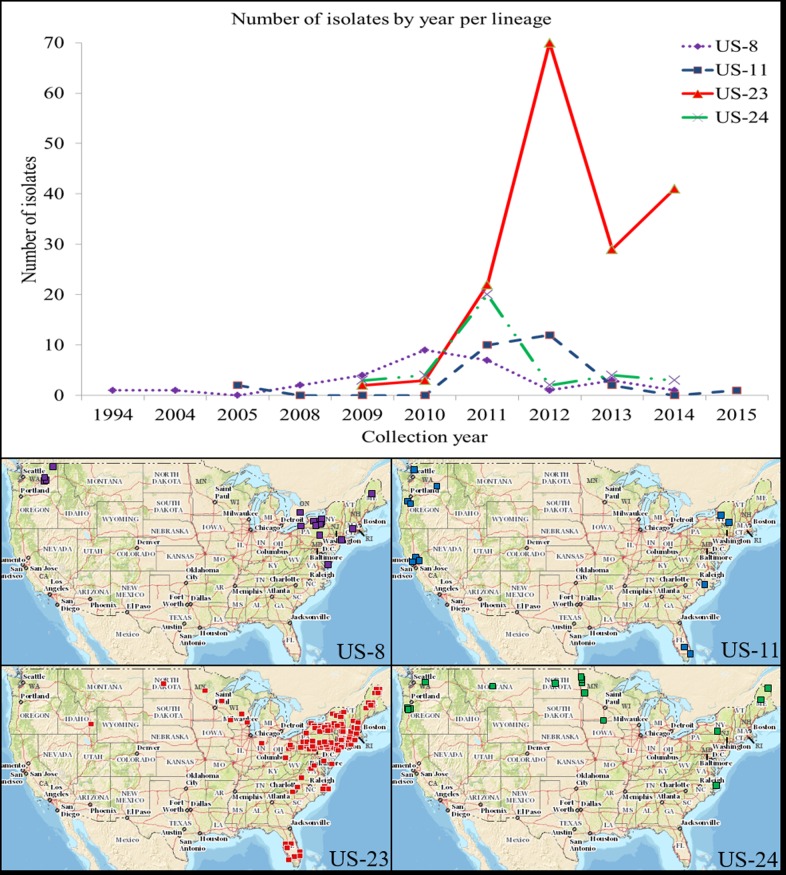
Number of isolates by year and collection location used in this study. Top: each line represents a lineage (dotted line [US-8, n = 29], dashed line [US-11, n = 27], solid line [US-23, n = 167], dotted line with dashes [US-24, n = 36]). Bottom: collection location of isolates by lineage. In some cases, map markers represent multiple isolates collected from that location.

Two hundred fifty-seven *P*. *infestans* isolates belonging to clonal lineages US-8, US-11, US-23, and US-24 were included in this study (S1 Table). United States isolates were collected from 23 states between 1994 and 2015, with an additional six isolates from Ontario, Canada included from 2010. The majority of isolates (94%) were collected from 2010 through 2014 (Fig 1, S2 Table).

Of the 257 isolates included in the study, 28 belonged to lineage US-8. These isolates were from seven US states (Idaho (ID), Massachusetts (MA), Maine (ME), New York (NY), Pennsylvania (PA), Virginia (VA), Washington (WA)) and Ontario, Canada, and were collected in 1994, 2004, and each year from 2008 to 2014 (Fig 1, S1 and S2 Tables). Twenty-seven isolates belonged to lineage US-11, originating from six US states (California (CA), Florida (FL), North Carolina (NC), NY, Oregon (OR), WA), and were collected in 2005, 2011, 2012, 2013, and 2015 (Fig 1, S1 and S2 Tables). Also included were thirty-six US-24 isolates collected from 2009 to 2014 from eight US states (ME, Minnesota (MN), Montana (MT), NC, North Dakota (ND), NY, OR, WA; Fig 1, S1 and S2 Tables). The largest group of isolates belonged to lineage US-23 (n = 166), and were from 19 US states (Connecticut (CT), Delaware (DE), FL, ID, Indiana (IN), MA, Maryland (MD), ME, MN, NC, ND, New Hampshire (NH), New Jersey (NJ), NY, Ohio (OH), PA, Rhode Island (RI), VA, Wisconsin (WI)), and were collected from 2009 to 2014 (Fig 1, S1 and S2 Tables). Lineage US-23 has been the predominant lineage in the United States since 2012, which is why it is the most-represented lineage in this study. A map of the contiguous Unites States with state labels is provided in S1 Fig.

### DNA extraction and genotyping-by-sequencing (GBS)

Two 5 mm stainless steel beads (Qiagen, Hilden, Germany) were added to each 2 ml round-bottom tube containing approximately 150 mg wet mycelia and run at 30 hz for 2 minutes using a Retsch MM400 Tissuelyser (Newton, PA). Extractions were then done using a DNeasy Plant Mini Kit (Qiagen) according to the manufacturer’s instructions. Prior to sample submission DNA quality was evaluated by gel electrophoresis, and DNA was quantified using a Qubit (Thermo Fisher Scientific, Waltham, MA). Following quality control checks, 30 μl of each DNA sample at 50–100 ng/μl were pipetted into 96 well plates (95 samples per plate plus one blank well), placed on ice, and immediately submitted to the Cornell University Institute for Genomic Diversity (IGD). Library preparation and GBS were done at the Cornell IGD as previously described [33]. Briefly, adapters were ligated to DNA samples following digestion by the restriction enzyme *Ape*KI [33]. Samples were then pooled, enriched by PCR, and purified prior to 100 bp single-end sequencing on an Illumina Hi-Seq 2500 (Illumina, San Diego, CA). All GBS data are available from the National Center for Biotechnology Information Sequence Read Archive (Accession number XXX available upon acceptance).

### Control isolates

Eight isolates were included as controls in the GBS analysis (Table 1). DNA was extracted from two separate mycelial samples from each of the control isolates, with the exception of isolate 11238 which had DNA extracted from three separate mycelial samples. DNA extractions were performed as described above. Replicated isolates were included in each of the three GBS sequencing runs (Table 1). For isolate 11238, aliquots of the same DNA extract as well as separate DNA extracts were included in the GBS analysis to evaluate error due to DNA extraction and DNA sequencing run.

**Table 1:** Replicated isolates included as controls in the GBS analysis, and average genetic distances among replicates and within their respective lineages.

Isolate name^w^	Genetic distance between replicates^x^	DNA extraction #^y^	GBS run #^z^
**Lineage US-23**			
11238_18	0.043	1	1
11238orgnl_90		1	2
11238orgnl_93		1	3
11238cntl1_88		2	2
11238cntl1_91		2	3
11238cntl2_89		3	2
11238cntl2_92		3	3
1726cntl22	0.042	1	3
1726cntl57		2	3
**Average genetic distance among replicates:**	0.043		
**Average genetic distance within entire lineage:**	0.089		
**Lineage US-8**			
1301cntl01	0.048	1	1
1301cntl94		2	1
1576cntlP2	0.088	1	2
1576cntlP3		2	3
824cntlP1	0.054	1	1
824cntlP2		2	2
2039cntlP1	0.085	1	1
2039cntlP2		2	2
**Average genetic distance among replicates:**	0.069		
**Average genetic distance within entire lineage:**	0.119		
**Lineage US-11**			
1310cntlP1	0.066	1	1
1310cntlP2		2	2
1403cntlP1	0.046	1	1
1403cntlP2		2	2
**Average genetic distance among replicates:**	0.056		
**Average genetic distance within entire lineage:**	0.095		

^w^ Isolate names beginning with the same 3 to 5 numbers are the same isolate, with the latter part of the isolate names identifying replicate information.

^x^ Values are pairwise genetic distances (Prevosti’s distance) between pairs of replicated samples. The value for isolate 11238 is the average of all pairwise genetic distances between seven replicates of isolate 11238.

^y^ Identical DNA extracts from the same isolate run as GBS replicates are indicated by the same DNA extraction number. Different DNA extracts from identical isolates are indicated by different DNA extraction numbers.

^z^ The study included a total of three 96-well GBS runs. Replicated control samples were included in each of the three runs.

### SNP calling and data filtering

Genotypes were called for all isolates simultaneously using the TASSEL 3.0.173 pipeline [40] which involved aligning barcoded reads (trimmed to 64 bp) to the *P*. *infestans* T30-4 reference genome assembly [41] in order to call SNPs. The Burrows-Wheeler aligner (bwa-aln and bwa-samse) with default parameters was used to align sequence tags to the reference genome [42]. Default parameters were otherwise used in TASSEL without imputation, with two exceptions: 1) Only sequence tags present >10 times were used to call SNPs; and 2) SNPs were output in variant call format (VCF) using the tbt2vcfplugin [40].

The resulting VCF file was filtered using VCFtools [43] on the Linux cluster at the Cornell University BioHPC Computing Lab. Individuals that failed to sequence were excluded from further analysis. Data were then separated into four VCF files according to *P*. *infestans* lineage. In each of the four VCF files bi-allelic SNPs were filtered to remove loci with minor allele frequency of less than 10%, mean site read depth of greater than 50, and greater than 20% missing data. Data were further filtered to a minimum genotype (site-by-individual) read depth of 7 using TASSEL [40].

### Data analysis

Principal component analysis (PCA) was done on the raw SNP data set containing all 257 isolates in TASSEL 5.0 (available at http://www.maizegenetics.net/#!tassel/c17q9) by converting genotypes to numeric scores and imputing missing data to the mean score for each site. Eigenvalues were imported into Microsoft Excel 2010 (Microsoft, Redmond, WA) to generate a scatter plot. Following confirmation of lineage assignments by PCA, the four filtered VCF files were used for within-lineage analyses in the R environment version 3.2.3 using the poppr [44] and adegenet [45] packages. Files were read using the function read.vcf and converted into genind or genclone objects with the functions vcfR2genind [46] and poppr::as.genclone (part of the poppr package), respectively. Using genind objects, neighbor-joining trees [47] were generated for each of the four lineages using the aboot function with 1,000 bootstrap replicates. Prevosti’s distance (prevosti.dist) [48], which is based on the fraction of different sites between samples, was chosen for its ability to handle missing data where missing data are considered equivalent in a given comparison [49]. Prevosti’s distance matrices were also used to calculate average genetic distances within each lineage and within replicated samples. A second set of trees was generated using Nei’s standard genetic distance (nei.dist) [50]. Each pair of trees per lineage was compared for consistency. Trees were formatted using Fig Tree version 1.4.2 (available at http://tree.bio.ed.ac.uk/software/figtree/). Additionally, K-means hierarchical clustering was done on each lineage as another way of assessing population structure [51]. The find.clusters function in the adegenet package [45] was used on genclone objects to determine the optimal number of clusters for each lineage based on Bayesian Information Criterion (BIC).

## Results

### GBS summary data and principal component analysis

There were a total of 243,981 SNPs in the unfiltered data set containing all four lineages. After filtering each single-lineage-VCF file for missing reads, mean site read depth of 7 and locus-by-individual (genotype) read depth of 7, the following number of SNPs were retained for each lineage: US-8 (3,774 SNPs); US-11 (4,363 SNPs); US-23 (5,070 SNPs); US-24 (4,353 SNPs). The frequency of heterozygous SNPs for each aforementioned lineage was 71%, 76%, 76%, and 75%, respectively. Based on Prevosti’s distance matrix, the average genetic distance within each lineage was 0.119 (US-8), 0.095 (US-11), 0.089 (US-23), and 0.102 (US-24). The average genetic distance between all replicated control samples was 0.047. None of the isolates in the study shared identical genotypes, including replicated controls. Variation within lineage and among technical replications is expected when using GBS [34].

**Fig 2:**
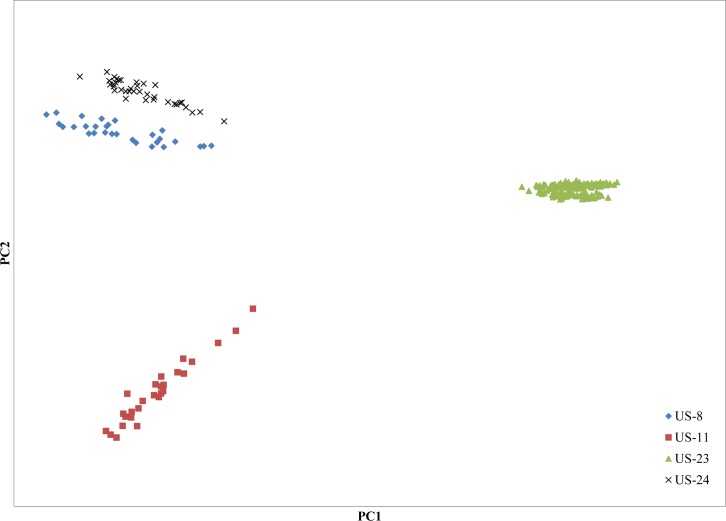
Principal component analysis of all 257 isolates used in the GBS study. Lineages are indicated by diamonds (US-8), squares (US-11), triangles (US-23), and x marks (US-24).

Each isolate used in this study had previously been assigned to a clonal lineage based on microsatellite genotyping. Separation of all four lineages was achieved using principal component analysis (PCA) on the raw GBS data set containing all 243,981 SNPs and all 257 isolates (Fig 2). All isolates were placed into one of four PCA groups corresponding to each of the four lineages. Principal components 1 and 2 collectively explained 21% of the variance in the data. Lineages US-11 and US-23 showed clear separation from each other and from US-8 and US-24. The latter two lineages were clearly separated, although to a lesser extent than US-11 and US-23 (Fig 2).

### Lineage US-8

**Fig 3:**
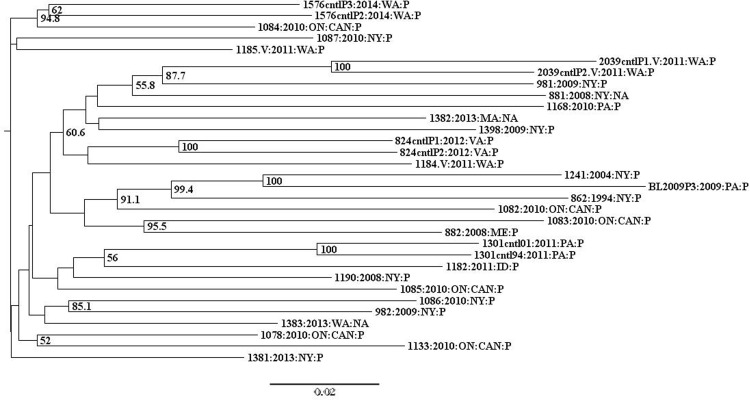
Neighbor-joining tree of US-8 isolates. Bootstrap values below 50% are not shown. Taxa are labeled by isolate code: collection year: collection state: host (P = potato, NA = information not available). Isolates that showed variation in their SSR profile are indicated by.V following their isolate code. Technical replicates are indicated by cntl (for control) and an additional sample identifier following their isolate code. Isolates from Ontario Canada are labeled ON:CAN.

K-means hierarchical clustering did not reveal grouping in the US-8 data, indicated by an optimal number of clusters of one. Neighbor-joining analysis of US-8 isolates revealed a broad distribution of isolates by geographic origin and collection date (Fig 3). The majority (82%) of US-8 isolates were collected in four states or provinces (NY (32%), PA (11%), WA (18%), and Ontario, Canada (21%)) (Fig 1, S1 and S2 Tables). One isolate from each of the following states made up the remainder of US-8 isolates: ME, MA, VA, ID, and OR. Some isolates grouped together by geographic origin in the NJ tree, such as two NY isolates (982 and 1086) and two isolates from ON, Can (1078 and 1133). There were also isolates from distant geographic regions that grouped together, like isolates from WA and ON, Can (1576 and 1084) and ID and PA (1182 and 1301) (Fig 3). Although many isolates were collected from the same state, only in a minority of cases did isolates group together by geographic origin.

The US-8 NJ tree was also evaluated by collection year. Eighty-six percent of US-8 isolates were collected in five years (2008–2011 and 2013) (Fig 1, S1 and S2 Tables). One isolate from each of the following years made up the remainder of US-8 isolates; 1994, 2004, 2012, and 2014. Isolates collected during the same year or sequential years did not group together overall (Fig 3). Three US-8 isolates deviated from the typical US-8 microsatellite genotype, and were denoted by.V following their isolate name. Two of these isolates (1184 and 2039) shared the same variant allele at marker Pi89. The third isolate (1185) had a unique variant allele at the same marker (S3 Table). None of the three SSR-variant isolates grouped together but all clustered within the US-8 lineage. Additionally, four US-8 isolates were replicated once each and included as controls (1576, 2039, 824, 1301, two samples each). The average genetic distance between replicated samples was 0.069, compared to an average distance of 0.119 for all US-8 isolates (Table 1). Each of the four control isolates grouped together with their replicated sample (Fig 3). A second NJ tree was constructed using Nei’s genetic distance to check the robustness of the analysis to different distance metrics. Both Prevosti’s and Nei’s trees had very similar overall topologies (data not shown).

### Lineage US-11

**Fig 4:**
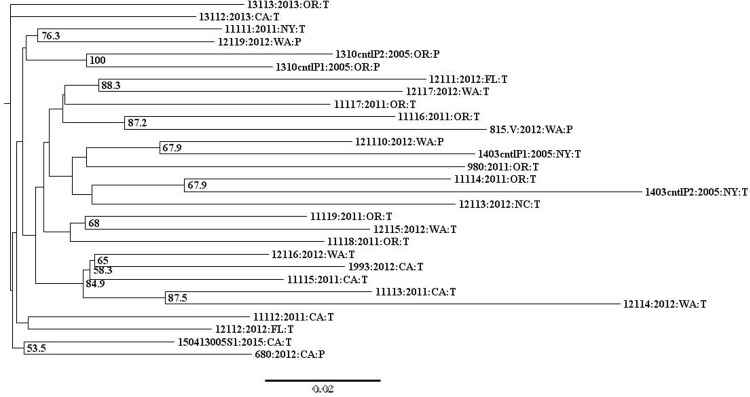
Neighbor-joining tree of US-11 isolates. Bootstrap values below 50% are not shown. Taxa are labeled by isolate code: collection year: collection state: host (P = potato, T = tomato). Isolates that showed variation in their SSR profile are indicated by.V following their isolate code. Technical replicates are indicated by cntl (for control) and an additional sample identifier following their isolate code

K-means hierarchical clustering did not reveal grouping in the US-11 data, indicated by an optimal number of clusters of one. Neighbor-joining analysis of US-11 isolates revealed a broad distribution of isolates by geographic origin and collection year (Fig 4) similar to what was observed with lineage US-8. Eighty-two percent of US-11 isolates were collected in three states (CA (26%), OR (30%), and WA (26%)). The remaining isolates were collected in FL (n = 2), NC (n = 1), and NY (n = 2) (Fig 1, S1 and S2 Tables). Some isolates grouped together by geographic origin in the NJ tree, such as two CA isolates (680 and 150413005S1), and isolates from OR and WA ([11116 and 815] and [11119 and 12115]). There were also isolates collected from distant geographic origins that grouped together, like NY and WA (11111 and 12119) and FL and WA (12111 and 12117) (Fig 4). Overall, there was not a consistent pattern of isolates grouping together by geographic origin.

When the US-11 NJ tree was evaluated by collection year there was no consistent grouping observed. The majority of US-11 isolates were collected during 2011 (37%) and 2012 (44%). Overall 2011 and 2012 isolates were scattered throughout the tree and did not consistently group with like-years. The remaining isolates were collected in 2005 (n = 2), 2013 (n = 2) and 2015 (n = 1) (Fig 1, S1 and S2 Tables). The two 2005 isolates (1310 and 1403) did not group together, nor did the 2013 isolates (13113 and 13112), and isolates did not consistently group together by host (Fig 4).

Additionally, two US-11 isolates were replicated once each and included as controls (1310 and 1403, two samples each). The average genetic distance between replicated samples was 0.056, compared to a distance of 0.095 for all US-11 isolates (Table 1). The two 1310 replicates grouped together. The two 1403 replicates were part of a larger group of five isolates, but were not directly adjacent to each other (Fig 4). Both Prevosti’s and Nei’s NJ trees had very similar overall topologies (data not shown).

### Lineage US-24

**Fig 5:**
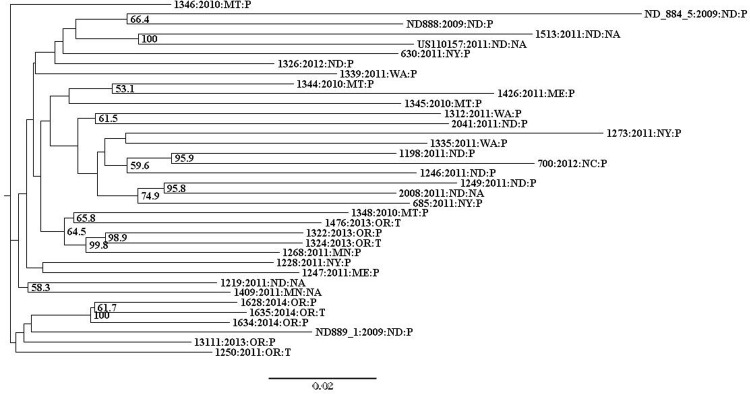
Neighbor-joining tree of US-24 isolates. Bootstrap values below 50% are not shown. Taxa are labeled by isolate code: collection year: collection state: host (P = potato, T = tomato, NA = information not available).

K-means hierarchical clustering did not reveal grouping in the US-24 data, indicated by an optimal number of clusters of one. Neighbor-joining analysis of US-24 isolates revealed a broad distribution of isolates by geographic origin and collection year (Fig 5). Fifty-five percent of US-24 isolates were from ND (33%) and OR (22%). The remaining 45% of isolates were from WA, MT, MN, NY, ME, and NC, with one to four isolates included from each state (Fig 1, S1 and S2 Tables). There were some isolates that grouped together by geographic origin in the NJ tree. For example, several ND isolates ([ND884_5 and ND888] and [1513 and US110157]) grouped together and isolates from OR generally grouped with other OR isolates collected the same year. There were also isolates from distant states that grouped together, like WA and ND (1312 and 2041), and ND and NC (1198 and 700) (Fig 5).

Fifty-six percent of US-24 isolates were collected in 2011. The remaining 44% of isolates were collected between 2009 and 2014, with two to four isolates collected from each of those years (Fig 1, S1 and S2 Tables). Isolates that grouped together by year were also collected from the same state (two ND isolates [2009], three and four OR isolates [2013 and 2014, respectively]) (Fig 5). Isolates from 2010 and 2012 did not group together overall, even though all four 2010 isolates were collected from MT, and isolates did not consistently group together by host. Both Prevosti’s and Nei’s NJ trees (reproducibility check) had very similar overall topologies (data not shown).

### Lineage US-23

#### K-means hierarchical clustering

**Fig 6:**
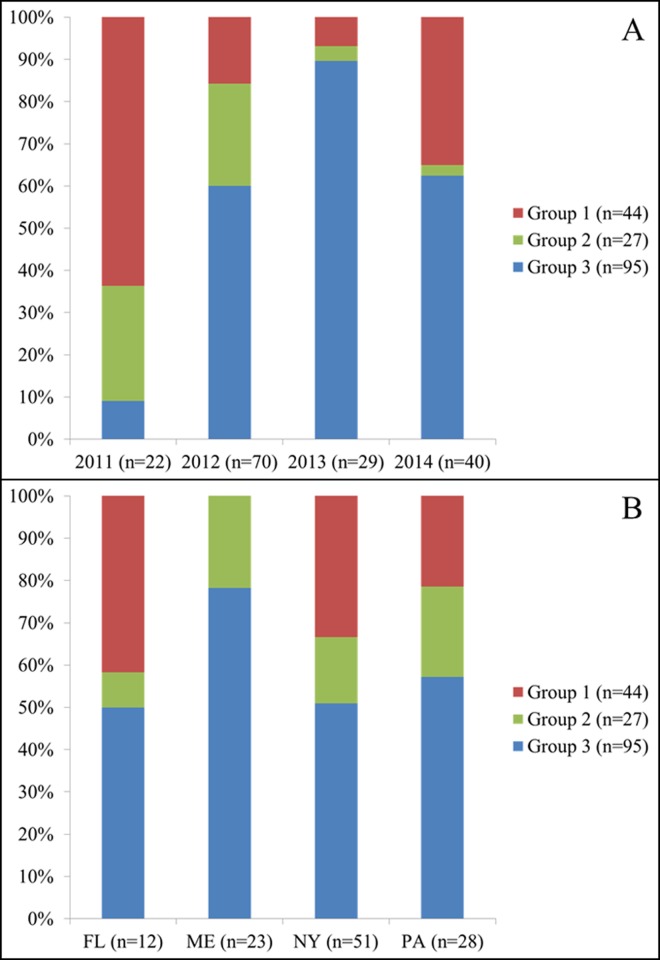
K-means hierarchical clustering of lineage US-23 revealed three clusters (group 1 [27% of isolates, n = 44]; group 2 [16% of isolates, n = 27]; group 3 [57% of isolates, n = 95]). **A.** Relative contribution of the four best-represented years (largest number of isolates) to each of the three K-means groups (group 1: top bars; group 2: middle bars; group 3: bottom bars). 97% of all US-23 isolates were collected from 2011 through 2014 (n = 161). **B.** Relative contribution of the four best-represented states (largest number of isolates) to each of the three K-means groups (group 1: top bars; group 2: middle bars; group 3: bottom bars). 69% of all US-23 isolates were from FL, ME, NY, and PA (n = 114).

US-23 individuals clustered into three groups based on K-means hierarchical clustering (Fig 6 and S1 Fig). Group 1 contained 44 individuals (27% of total), group 2 contained 27 individuals (16% of total), and group 3 contained 95 individuals (57% of total). K-means groups 1 and 3 were not closely associated with NJ clusters, however the majority of group 2 isolates clustered together (S2 Fig). Fig 6 shows a breakdown of the relative proportion of each K-means group by year and state, including the four most-sampled years (2011–2014, 97% of total) and states (FL, ME, NY, PA, 69% of total). A random distribution of isolates into each of the three groups would have resulted in approximately equally-sized bars for each group across years and states. Fig 6A shows significant variability in the relative contribution of each of the four years to each of the three K-means groups. For example, 9% of 2011 isolates belonged to group 3, which contained 57% of all US-23 isolates. Similarly, 64% of 2011 isolates belonged to group 1, which contained a comparatively small 27% of all US-23 isolates. Group 2 was heavily weighted towards years 2011 and 2012. There was less variability in the relative contribution of each of the four states to each of the three groups, with the exception of ME isolates which clustered only into groups 2 and 3 (Fig 6B).

#### Neighbor-joining analysis

**Fig 7:**
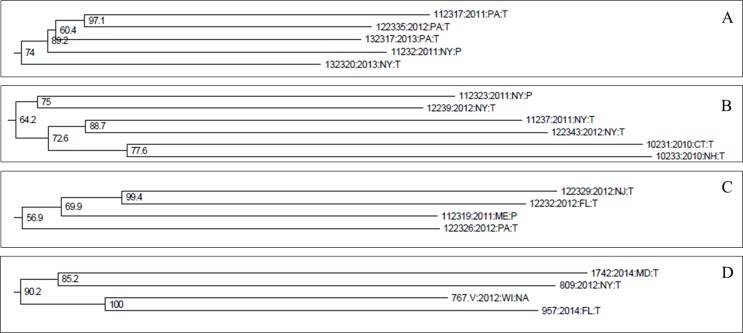
**Representative examples of US-23 isolates that grouped together by geographic origin (A and B), and US-23 isolates from distant geographic origins that grouped together (C and D).** All four groups shown here are exactly as they appear in the US-23 neighbor-joining tree containing all 166 US-23 isolates (S3 Fig).

The average genetic distance between isolates within lineage US-23 was 0.089. Sixty-nine percent of US-23 isolates were from FL (7%), ME (14%), NY (31%) and PA (17%). The remaining 31% of isolates were from 15 states from the east coast to as far west as ID, with one to nine isolates included from each state (Fig 1, S1 and S2 Tables). There were examples of isolates that grouped together by geographic regions, like the group shown in Fig 7A which contained three isolates from PA and two isolates from NY. Similarly, Fig 7B shows four isolates from NY and two isolates each from CT, NH and PA. There were also numerous examples of isolates that grouped together from distant states, like the group shown in Fig 7C which contained one isolate each from NJ, FL, ME and PA. Similarly, Fig 7D shows a group containing one isolate each from NY, MD, WI, and FL. Although many US-23 isolates were collected from the same state they did not group together consistently by geographic origin overall, nor was there consistent grouping by host (S3 Fig).

**Fig 8:**
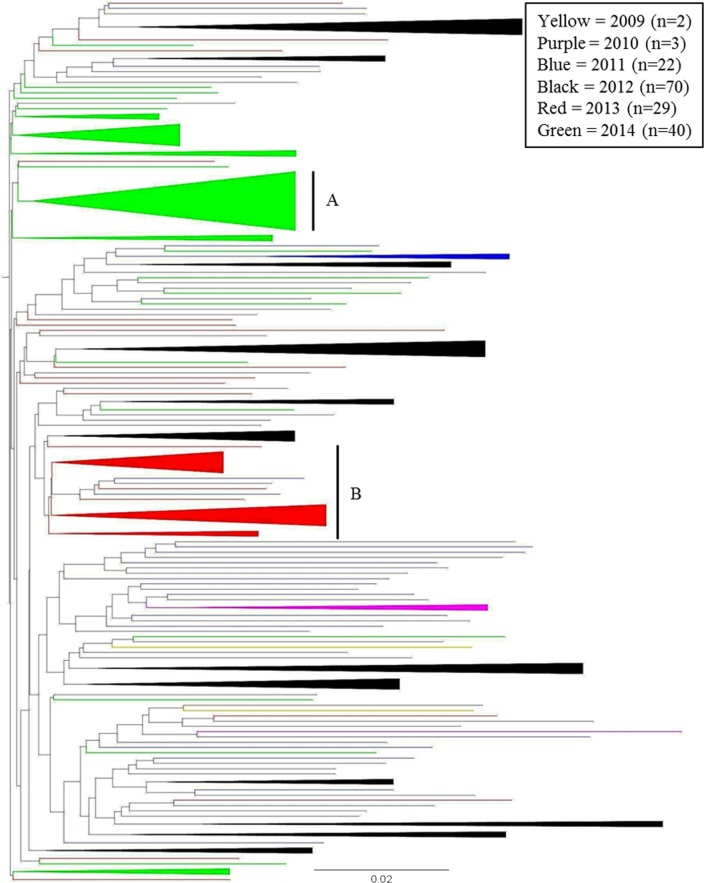
Neighbor-joining tree of all 166 US-23 individuals, color-coded by collection year. 97% of US-23 isolates were collected from 2011 through 2014. Isolates collected in 2011 (blue) and 2012 (black) are consistently scattered throughout the tree. Isolates collected in 2013 (red) and 2014 (green) group strongly together. **A.** Group of 11 isolates collected in 2014 from six states (MA, NY, VA, NC, FL, and ID). **B.** Group of 17 isolates, 14 of which were collected in 2013 from eight states (ME, RI, MA, NY, PA, OH, IN, and WI). The remaining three isolates in this group were collected in 2011 (NY and PA) and 2012 (PA).

The US-23 NJ tree had significantly more grouping by collection year than by geographic origin (Fig 8). Ninety-seven percent of US-23 isolates were collected from 2011 through 2014 (2011 (13%), 2012 (42%), 2013 (17%), 2014 (24%)). The remaining three percent of isolates were collected in 2009 (n = 2) and 2010 (n = 3) (Fig 1, S1 and S2 Tables). The two 2009 isolates did not group together. There was a group of 18 isolates which, besides two 2010 isolates, were all collected in 2011 and 2012 (S3 Fig). Two notable groups of isolates that clustered by collection year are indicated in Fig 8. The letter A in Fig 8 designates a group of 11 isolates, all of which were collected in 2014. The isolates shown in Fig 8A came from MA, NY, VA, NC, FL, and ID. The letter B in Fig 8 designates a group of 17 isolates, 14 of which were collected in 2013 from nine states (ME, RI, MA, NY, PA, NC, OH, IN, and WI) and three of which were collected in 2011 (NY and PA) and 2012 (PA).

Two US-23 isolates were replicated and included as controls (1726 and 11238). Isolate 1726 was replicated once (two samples total), and isolate 11238 was replicated six times (seven samples total) (Table 1). The 1726 replicates were part of a larger group of 12 isolates, but were not directly adjacent to each other on the NJ tree. All 11238 replicates were adjacent to each other on the NJ tree (S3 Fig). The average genetic distance between replicated US-23 samples was 0.043, compared to 0.089 for all US-23 isolates (Table 1). The average genetic distance between 11238 replicates of the same DNA extract run on different GBS plates was 0.043, and the average genetic distance between replicates of different DNA extracts run on the same plate was 0.041. Additionally, both Prevosti’s and Nei’s NJ trees (reproducibility check) had very similar overall topologies (data not shown). Twenty-six US-23 isolates deviated from the typical US-23 SSR genotype at the same D13 marker, and were denoted by.V following their isolate name. Twenty-four of these isolates shared the same variant alleles at marker D13, and two isolates (isolates 122320 and 112312) each had unique alleles at this marker. Isolate 112312 also differed by two alleles at the G11 marker (S3 Table). Although 14 out of 26 SSR-variant isolates did group with other variants, overall they did not collectively group together and were scattered throughout the NJ tree (S3 Fig).

## Discussion

In this study we used genotyping-by-sequencing to identify diversity within four clonal lineages of *P*. *infestans*. This work builds on previous studies that identified variability among asexual progeny of *P*. *infestans* (Abu-El Samen et al., 2003a, 2003b; Caten and Jinks, 1968; Goodwin et al., 1995b; Miller et al., 1998; reviewed in Goodwin, 1997). For example, Abu-El Samen et al. (2003b) found significant variability in virulence among asexual progeny when potato differentials were inoculated with 102 single-zoospore isolates derived from five different parental isolates. The two parental isolates showing the lowest and highest levels of phenotypic diversity among asexual progeny were chosen for genotypic analysis using random amplified polymorphic DNA (RAPD) and amplified fragment length polymorphism (AFLP) [32]. Significant genotypic variability, presumed to be the result of mutation and mitotic recombination, was observed among the progeny of both parental isolates, but was not well-correlated with phenotypic variability. Studies like these were important in demonstrating that, with sufficient genetic marker density, variability in asexual progeny of *P*. *infestans* could be detected. Our goal was to use GBS to generate a large number of genetic markers to evaluate genetic diversity within clonal lineages of *P*. *infestans*, and to use those data to detect sub-lineages within a naturally-occurring asexual population.

Using GBS, we identified between 3,774 and 5,070 SNPs within lineages US-8, US-11, US-23, and US-24 and found that principal component analysis (PCA) could separate all isolates into their respective lineages. The relatively close grouping of lineages US-8 and US-24 in Fig 2 compared to the other lineages is consistent with previous findings [8]. To investigate population sub-structuring and inoculum dispersal patterns, pairwise distances between all isolates within each lineage were calculated using Prevosti’s genetic distance [48], and the resulting distance matrices were used to construct NJ trees [47,52]. Prevosti’s genetic distance was also compared with Nei’s genetic distance because both rely on allele frequencies to determine distances between individuals [48,50]. This approach, rather than relying on multi-locus genotype frequencies, was appropriate for assessing diversity in our data because each individual was genetically unique. Additionally, K-means hierarchical clustering identified three groups within lineage US-23. The lower sample sizes of lineages US-8, US-11, and US-24 compared to US-23 likely contributed to our inability to identify K-means groups within those lineages.

The average genetic distance within each lineage was 0.119 (US-8), 0.095 (US-11), 0.089 (US-23), and 0.102 (US-24). We hypothesized that the older US-8 and US-11 lineages, first identified in 1992 and 1994, respectively [11], would have greater average genetic distances than the younger US-23 and US-24 lineages, first identified in 2009 [13], due to the accumulation of mutations over time. This phenomenon has been well documented in the US-1 lineage of *P*. *infestans*, which was the globally-predominant lineage in the mid to late 20^th^ century (reviewed by Goodwin (1997)). An important caveat to consider is the fact that genotypic diversity is expected to increase with sample size [53,54]. Lineages US-8 and US-11 had the smallest sample sizes (n = 28 and n = 27, respectively), followed by US-24 (n = 38) and US-23 (n = 166). Despite having the largest sample size (more than 4.5 times larger than the second-most-sampled lineage) US-23 had the lowest average genetic distance among isolates. This supports our hypothesis while considering the effect of uneven sample sizes. The result was less clear for lineage US-24, which had an average genetic distance that was lower than lineage US-8, but higher than lineage US-11. Although lineage US-24 was first reported in 2009 [13,15], as with all naturally-occurring lineages, its true age is not known. Therefore, it is possible that US-24 existed prior to 2009, thereby increasing the time during which mutations could have accumulated. Additionally, genetic drift resulting from annual genetic bottlenecks caused by loss of host tissue and winter-killing of the pathogen could have reduced diversity within lineage US-11 [53]. These questions warrant further investigation.

*Phytophthora infestans* is known to move locally and regionally by wind-dispersal [55] and both regionally and nationally through the shipment of infected seed tubers and tomato transplants [13]. Through the analyses of the US-8, US-11, and US-24 NJ trees we found examples of isolates collected from the same state during the same year that grouped together, like US-24 isolates from 2009 (ND), 2013 (OR), and 2014 (OR). The ND isolates were both from Grand Forks, and the OR isolates were from Philomath, Corvallis, and Lebanon which are less than 50 miles apart, which is a feasible distance for an individual to spread by wind in a single season [18,55]. However, the grouping of these isolates could also be explained by the transport of common inoculum on infected plant material to each collection site.

There were also isolates collected from distant states during the same year that grouped together, like US-8 isolates from ID and PA in 2011 (Fig 3), US-11 isolates from FL and WA in 2012 (Fig 4), and US-24 isolates from ND and NY in 2011 (Fig 5). The grouping of these isolates supports long-distance pathogen transport on infected host tissue, as the spread of airborne *P*. *infestans* inoculum over such distances in a single season is highly unlikely. Certified seed potatoes are produced in fifteen US states with Idaho (29%) and North Dakota (15%) accounting for the largest proportions of production, followed by Colorado, Maine, Montana, and Wisconsin each with approximately 10% of the total production (USDA, NASS 2015). Little information is available on where seed potatoes are shipped for production, but several states with commercial potato production have limited or no certified seed production, suggesting that seeds are likely shipped from state to state (USDA, NASS 2015). Overall, US-8, US-11, and US-24 isolates did not group by geographic origin, which may be evidence that individuals regularly moved throughout the sampling area by infected seed tubers or tomato seedlings to initiate infections. Alternatively, this might indicate that enough individuals are overwintering in each state, presumably in cull piles or as unharvested tubers, so that the majority of isolates collected from a given state are members of separate sub-lineages rather than descendants of the same aerially-dispersed sub-lineage. This scenario may explain some of the cases where isolates from the same state did not group together. Future work involving higher-density sampling in one or a few smaller geographic areas could help to address this question.

Similar to the analysis by geography, the US-8, US-11, and US-24 NJ trees did not show consistent groupings of individuals by collection year. The groupings of individuals by year that were observed in the US-24 NJ tree could be explained by transport of infected plant material or regional wind-dispersal because those isolates were also collected from the same state (2009, ND, n = 2; 2013, OR, n = 3; 2014, OR, n = 3).

Consistent with the US-8, US-11, and US-24 results, US-23 individuals were not significantly grouped by geographic origin (Fig 6 and S2 Fig). This result is exemplified by isolates 645 (tomato) and 122345 (potato), both of which were collected from the same Penn State research farm three weeks apart and resulted from natural inoculum. On the NJ tree these isolates did not group near each other, but isolate 122345 did group closely with another 2012 isolate collected on tomato from Troy, ME (isolate 122318) (S3 Fig). There were examples of individuals from the same state that grouped together from the same year (ME, 2012, n = 2) and from different years (NY, 2012 and 2013, n = 2). The former could reflect regional pathogen spread by wind dispersal, while the latter could reflect pathogen overwintering in potato cull piles or unharvested tubers. However, there was not a consistent pattern of isolates grouping by geographic origin overall.

Contrary to results from the US-8, US-11, and US-24 NJ trees, some US-23 individuals did group together significantly by collection year (Figs 6, 7 and 8). In particular, isolates from 2013 and 2014 showed a strong tendency to group with other isolates from those years. Given the large geographic areas represented in the major 2013 and 2014 groups (Fig 8), this is a strong indication that long-distance pathogen transport by infected host plant material played a significant role in initiating late blight epidemics in those years. The late blight pandemic in the eastern United States in 2009 [13] illustrated how efficiently *P*. *infestans* can be dispersed over large distances through the movement of infected plant material. During that outbreak, late blight-infected tomato seedlings, which were observed by plant pathologists at numerous large retail garden centers throughout the Northeast, were identified as the primary source of inoculum. This was unusual because the source of late blight inoculum was observable, compared to typical late blight outbreaks where the source of primary inoculum is ambiguous (infected seed tubers, volunteer potatoes and tomatoes, potato cull piles, etc.). Although late blight has recurred each year since the 2009 outbreak, the widespread distribution of infected tomato seedlings is not known to have re-occurred in the United States.

Individuals within each lineage shared microsatellite genotypes with the exception of three US-8 individuals, one US-11 individual, and 26 US-23 individuals. These exceptions were variants within each clonal lineage where one or two of the twelve microsatellite loci were variant from the standard genotype for that lineage (S3 Table). The lack of grouping of US-8 and US-23 microsatellite variants on the NJ trees may indicate homoplasy at the variant microsatellite loci. Such homoplasy would be much less likely at the numerous SNP sites scattered throughout the genome used to construct the NJ trees.

Replicated control samples were also included in each of the three GBS runs to assess experimental error, such as sequencing error, technical error during restriction and ligation, and DNA extraction, which may have influenced our data. The average genetic distance between control samples was consistently approximately half that of the average distance within the entire lineage. This pattern was consistent whether the exact same DNA extract was replicated on different GBS plates, or separate DNA extracts of the same isolate were run on the same GBS plate. This indicates that part of the genetic distance separating replicated controls is probably the result of sequencing, ligation and barcoding error, and not differences in the sample DNA. Seventeen out of 21 replicated samples were adjacent to their replicate in the NJ trees. There were two replicated control isolates (1403 [US-11, n = 2] and 1726 [US-23, n = 2]) that were not directly adjacent to each other on the NJ tree, although they were relatively near to each other. All replicated controls, except the robustly-replicated US-23 isolate 11238, were stored in separate long-term storage vials. It is possible that the length of time in culture prior to storage and/or the number of culture transfers may have resulted in genotypic variation that was observed in this study. We were not able to differentiate real differences in replicated sample DNA versus sequencing error. Regardless, the consistency with which our replicated control samples grouped together on the NJ trees, along with the significantly lower average genetic distances between controls compared to entire lineages, gives us confidence that experimental error did not significantly influence our interpretations.

Here, we showed that there is a significant amount of genetic diversity within clonal lineages of *P*. *infestans*, which is consistent with results from previous studies. Additionally, our data indicate that GBS is capable of generating enough genetic markers to detect sub-structuring within naturally-occurring clonal populations. Our analyses revealed that long-distance pathogen transport, presumably by infected plant tissue, plays an important role in initiating late blight outbreaks on an annual basis. This highlights an opportunity for improving late blight management, and warns of the potential for rapid long-distance dispersal of novel *P*. *infestans* genotypes.

## Supporting Information

S1 Fig A map of the contiguous United States with state labels. (TIF)

S2 Fig Neighbor joining tree of clonal lineage US-23, based on Prevosti’s distance, color-coded by K-means hierarchical clustering groups (group 1 [27% of isolates, n = 44, red]; group 2 [16% of isolates, n = 27, green]; group 3 [57% of isolates, n = 95, blue]). Groups 1 and 3 do not associate well with the neighbor-joining groups, which is evidence for panmixia in a sexual population, or individuals moving throughout the sampling area in an asexual population. Group 2 does associate well with a neighbor-joining group, which is evidence for population sub-structuring. Replicated control isolates were excluded from the analysis to avoid biasing K-means results. S1 and S2 Figs were generated by separate NJ algorithm runs, therefore some branch arrangements differ between the two NJ trees.
(TIF)

S3 Fig Neighbor-joining tree of US-23 isolates. Bootstrap values below 50% are not shown. Taxa are labeled by isolate code: collection year: collection state: host (P = potato, T = tomato, NA = information not available). Isolates that showed variation in their SSR profile are indicated by.V following their isolate code. Technical replicates included isolate 1726 (replicated once) and isolate 11238 (replicated six times).
(TIF)

S1 Table All isolates included in the GBS study sorted by clonal lineage and collection location. (DOCX)

S2 Table Number of *P*. *infestans* isolates within each lineage organized by year and collection location. (DOCX)

S3 Table Allele sizes for eleven SSR markers used to assign isolates to clonal lineages. (DOCX)
