# Supplementary figures and images for: Genetic Variation within Clonal Lineages of Phytophthora infestans Revealed through Genotyping-By-Sequencing, and Implications for Late Blight Epidemiology
Source: PLoS One. 2016 Nov 3;11(11):e0165690. doi: 10.1371/journal.pone.0165690 (PMC5094694; doi:10.1371/journal.pone.0165690)

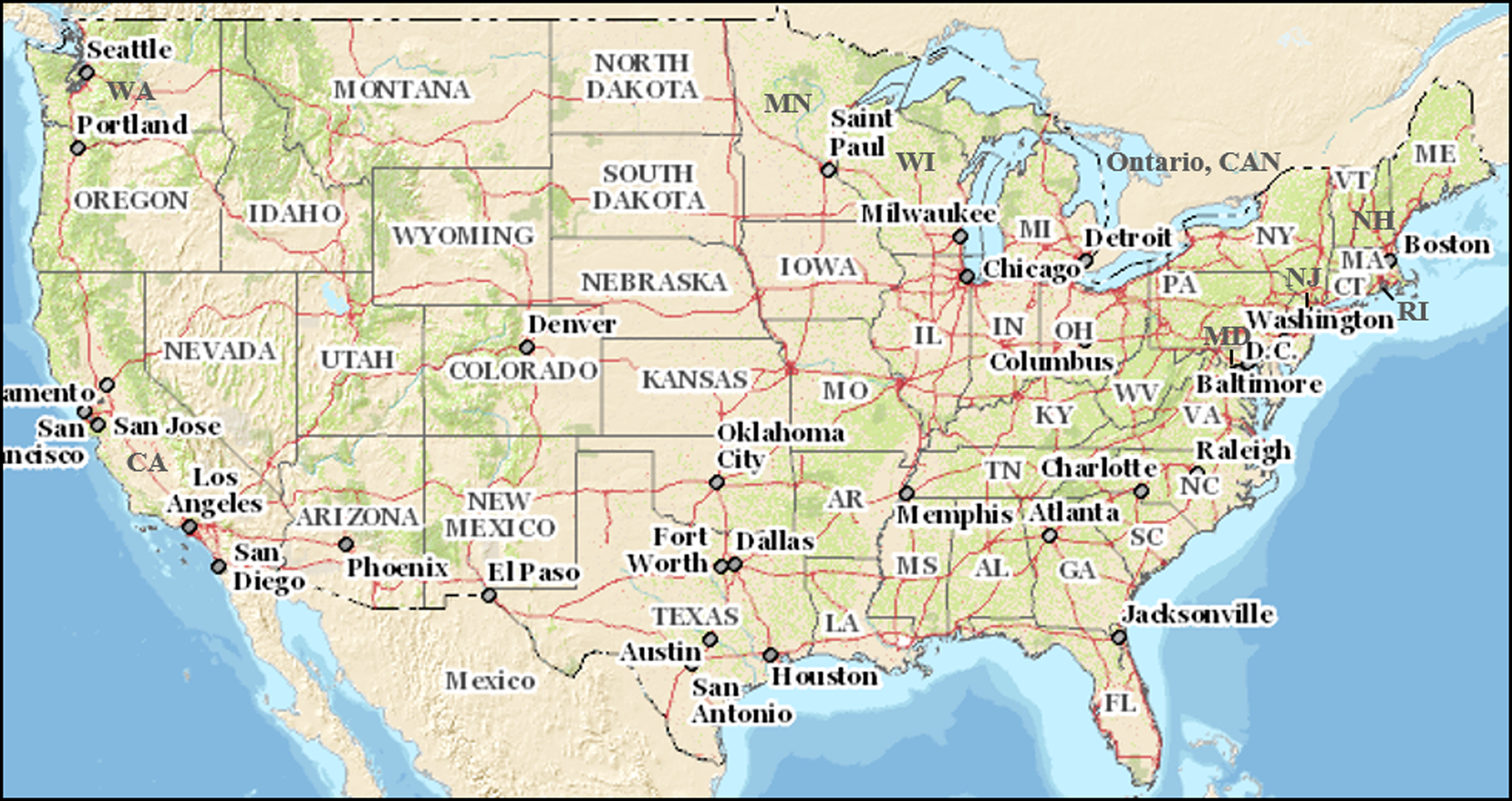

Supplement: S1 Fig — (TIF) [file pone.0165690.s001.tif]

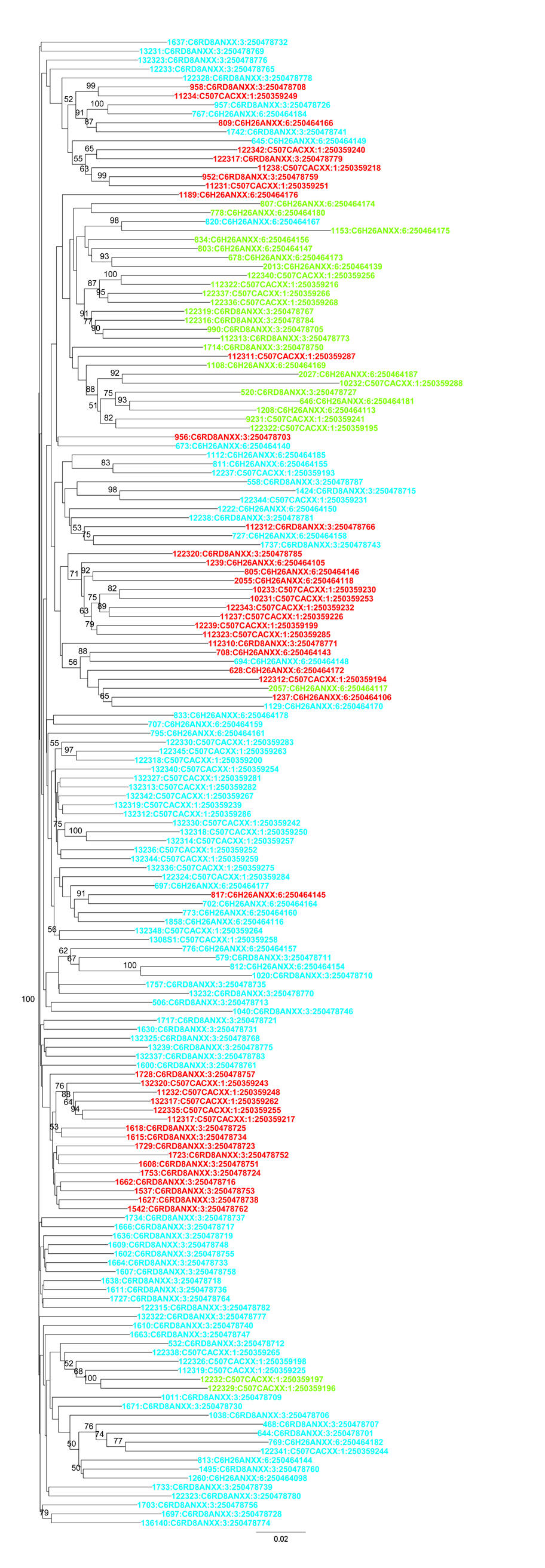

Supplement: S2 Fig — Groups 1 and 3 do not associate well with the neighbor-joining groups, which is evidence for panmixia in a sexual population, or individuals moving throughout the sampling area in an asexual population. Group 2 does associate well with a neighbor-joining group, which is evidence for population sub-structuring. Replicated control isolates were excluded from the analysis to avoid biasing K-means results. S1 and S2 Figs were generated by separate NJ algorithm runs, therefore some branch arrangements differ between the two NJ trees. (TIF) [file pone.0165690.s002.tif]

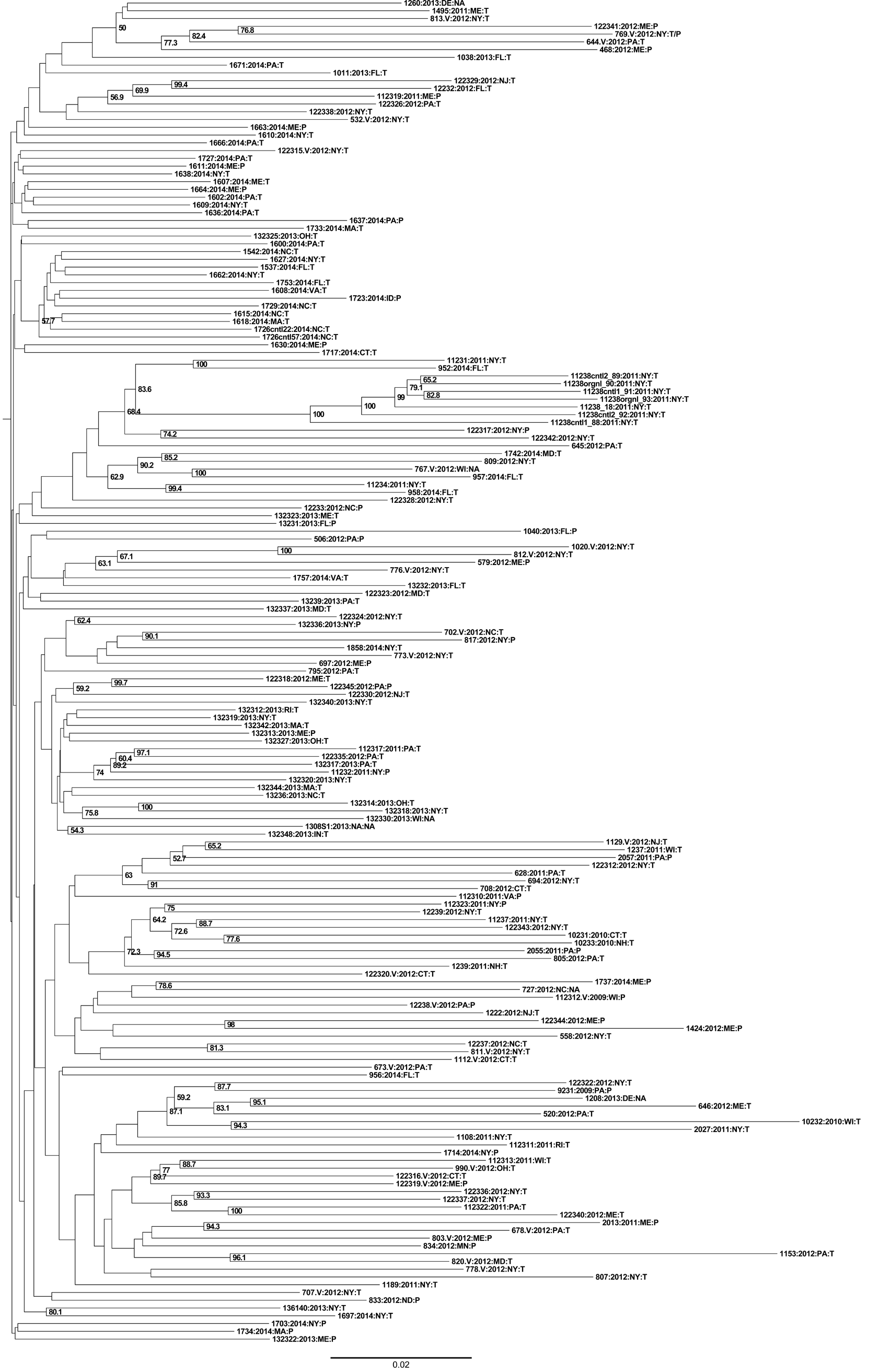

Supplement: S3 Fig — Bootstrap values below 50% are not shown. Taxa are labeled by isolate code: collection year: collection state: host (P = potato, T = tomato, NA = information not available). Isolates that showed variation in their SSR profile are indicated by.V following their isolate code. Technical replicates included isolate 1726 (replicated once) and isolate 11238 (replicated six times). (TIF) [file pone.0165690.s003.tif]
