# Supplementary material for: Genetic Variation within Clonal Lineages of Phytophthora infestans Revealed through Genotyping-By-Sequencing, and Implications for Late Blight Epidemiology
Source: PLoS One. 2016 Nov 3;11(11):e0165690. doi: 10.1371/journal.pone.0165690 (PMC5094694; doi:10.1371/journal.pone.0165690)
Supplement: S1 Table — (DOCX) [file pone.0165690.s004.docx]

**S1 Table.** All isolates included in the GBS study sorted by clonal lineage and collection location.

| **Isolate name** | **USAblight ID^x^** | **Clonal lineage^y^** | **Country** | **State or province** | **City or county** | **Geographic coordinates^z^** | **Host** | **Sample year** | **Date collected** |
| --- | --- | --- | --- | --- | --- | --- | --- | --- | --- |
| 1182 | na | us8 | USA | ID | Bonner Ferry Co | 48°41'30.3"N 116°19'02.7"W | P | 2011 | 10/6/2011 |
| 1382 | na | us8 | USA | MA | Hadley | 42°20'26.6"N 72°35'07.7"W | na | 2013 | 10/8/2013 |
| 882 | na | us8 | USA | ME | na | na | P | 2008 | 9/1/2008 |
| 862 | na | us8 | USA | NY | na | na | P | 1994 | na |
| 1241 | na | us8 | USA | NY | na | na | P | 2004 | na |
| 881 | na | us8 | USA | NY | Elba | 43°04'38.8"N 78°11'13.2"W | na | 2008 | 9/1/2008 |
| 1190 | na | us8 | USA | NY | Monroe Co | 43°09'40.5"N 77°36'54.3"W | P | 2008 | 9/1/2008 |
| 982 | na | us8 | USA | NY | Bliss | 42°34'38.6"N 78°15'09.3"W | P | 2009 | 9/1/2008 |
| 1398 | na | us8 | USA | NY | Freeville | 42°30'50.9"N 76°20'50.3"W | P | 2009 | 9/25/2009 |
| 981 | na | us8 | USA | NY | Steuben Co | 42°17'31.0"N 77°22'50.0"W | P | 2009 | 11/3/2011 |
| 1086 | na | us8 | USA | NY | Yates Co | 42°39'45.5"N 77°03'56.6"W | P | 2010 | 8/19/2010 |
| 1087 | na | us8 | USA | NY | Wayne Co | 43°09'15.9"N 77°01'02.9"W | P | 2010 | 9/15/2010 |
| 1381 | 130829203S1 | us8 | USA | NY | Erie Co | 42°42'37.4"N 78°39'46.2"W | P | 2013 | 8/30/2013 |
| 1078 | na | us8 | CAN | ON | Shelburne | 44°04'45.0"N 80°12'04.5"W | P | 2010 | 8/17/2010 |
| 1082 | na | us8 | CAN | ON | Shelburne | 44°04'45.0"N 80°12'04.5"W | P | 2010 | 8/17/2010 |
| 1083 | na | us8 | CAN | ON | Shelburne | 44°04'45.0"N 80°12'04.5"W | P | 2010 | 8/17/2010 |
| 1084 | na | us8 | CAN | ON | Shelburne | 44°04'45.0"N 80°12'04.5"W | P | 2010 | 8/17/2010 |
| 1085 | na | us8 | CAN | ON | Shelburne | 44°04'45.0"N 80°12'04.5"W | P | 2010 | 8/17/2010 |
| 1133 | na | us8 | CAN | ON | Shelburne | 44°04'45.0"N 80°12'04.5"W | P | 2010 | 8/17/2010 |
| BL2009P3 | na | us8 | USA | PA | Blair Co | 40°30'50.3"N 78°24'52.6"W | P | 2009 | na |
| 1168 | na | us8 | USA | PA | Erie Co | 42°42'37.4"N 78°39'46.2"W | P | 2010 | 8/17/2010 |
| 1301 | 110907001 | us8 | USA | PA | Erie Co | 42°42'37.4"N 78°39'46.2"W | P | 2011 | 9/7/2011 |
| 824 | 120613056S1 | us8 | USA | VA | Painter | 37°35'07.3"N 75°47'07.2"W | P | 2012 | 6/6/2012 |
| 1184 | na | us8.v | USA | WA | Pasco | 46°14'23.0"N 119°06'07.9"W | P | 2011 | 10/6/2011 |
| 2039 | na | us8.v | USA | WA | Pasco | 46°14'23.0"N 119°06'07.9"W | P | 2011 | 10/6/2011 |
| 1185 | 111011001 | us8.v | USA | WA | Othello | 46°49'30.4"N 119°10'30.6"W | P | 2011 | 10/11/2011 |
| 1383 | na | us8 | USA | WA | Columbia Basin | 46°47'18.9"N 119°18'28.3"W | na | 2013 | 10/14/2013 |
| 1576 | na | us8 | USA | WA | Eltopia | 46°27'33.6"N 119°01'00.8"W | P | 2014 | na |
| 11112 | 110817001 | us11 | USA | CA | Stockton | 37°57'31.9"N 121°17'42.1"W | T | 2011 | 8/17/2011 |
| 11113 | 110829001 | us11 | USA | CA | Stockton | 37°57'31.9"N 121°17'42.1"W | T | 2011 | 8/29/2011 |
| 11115 | na | us11 | USA | CA | San Joaquin Co | 37°57'31.1"N 121°16'48.5"W | T | 2011 | 12/1/2011 |
| 680 | 120702103S1 | us11 | USA | CA | San Francisco Co | 37°46'28.2"N 122°26'25.3"W | P | 2012 | 7/2/2012 |
| 1993 | 120702104S1 | us11 | USA | CA | San Francisco | 37°46'28.2"N 122°26'25.3"W | T | 2012 | 7/2/2012 |
| 13112 | 131011282S1 | us11 | USA | CA | San Francisco | 37°46'28.2"N 122°26'25.3"W | T | 2013 | 10/16/2013 |
| 150413005S1 | 150413005S1 | us11 | USA | CA | Woodland | 38°40'44.9"N 121°46'28.9"W | T | 2015 | 4/13/2015 |
| 12111 | 120613048S1 | us11 | USA | FL | Loxahatchee | 26°46'21.8"N 80°14'21.1"W | T | 2012 | 4/28/2012 |
| 12112 | 120613049S1 | us11 | USA | FL | Collier Co | 26°14'52.4"N 81°35'29.2"W | T | 2012 | 5/4/2012 |
| 12113 | 120705112S1 | us11 | USA | NC | na | na | T | 2012 | 7/6/2012 |
| 1403 | na | us11 | USA | NY | Albany | 42°39'05.2"N 73°45'43.1"W | T | 2005 | na |
| 11111 | 110818001 | us11 | USA | NY | Boonville | 43°28'59.9"N 75°20'17.6"W | T | 2011 | 8/18/2011 |
| 1310 | na | us11 | USA | OR | na | na | P | 2005 | na |
| 980 | 110830003 | us11 | USA | OR | Corvallis | 44°33'54.5"N 123°16'01.4"W | T | 2011 | 8/30/2011 |
| 11114 | 110830002 | us11 | USA | OR | Corvallis | 44°33'54.5"N 123°16'01.4"W | T | 2011 | 8/30/2011 |
| 11116 | na | us11 | USA | OR | Linn Co | 44°32'03.3"N 122°46'07.1"W | T | 2011 | 10/20/2011 |
| 11117 | na | us11 | USA | OR | Linn Co | 44°32'03.3"N 122°46'07.1"W | T | 2011 | 10/20/2011 |
| 11118 | na | us11 | USA | OR | Linn Co | 44°32'03.3"N 122°46'07.1"W | T | 2011 | 10/20/2011 |
| 11119 | na | us11 | USA | OR | Linn Co | 44°32'03.3"N 122°46'07.1"W | T | 2011 | 10/20/2011 |
| 13113 | 131012284S1 | us11 | USA | OR | Corvallis | 44°33'54.5"N 123°16'01.4"W | T | 2013 | 10/16/2013 |
| 12114 | 121119306S2 | us11 | USA | WA | Skagit Co | 48°26'09.5"N 122°25'40.2"W | T | 2012 | 11/21/2012 |
| 12115 | 121120309S1 | us11 | USA | WA | Skagit Co | 48°26'09.5"N 122°25'40.2"W | T | 2012 | 11/21/2012 |
| 12116 | 121120310S1 | us11 | USA | WA | Skagit Co | 48°26'09.5"N 122°25'40.2"W | T | 2012 | 11/21/2012 |
| 12117 | 121120311S1 | us11 | USA | WA | Skagit Co | 48°26'09.5"N 122°25'40.2"W | T | 2012 | 11/21/2012 |
| 12119 | 121120312S1 | us11 | USA | WA | Skagit Co | 48°26'09.5"N 122°25'40.2"W | P | 2012 | 11/21/2012 |
| 121110 | na | us11 | USA | WA | Franklin Co | 46°32'47.8"N 118°55'21.1"W | P | 2012 | 11/21/2012 |
| 815 | 121119307S2 | us11.v | USA | WA | Skagit Co | 48°26'09.5"N 122°25'40.2"W | P | 2012 | 11/21/2012 |
| 10231 | na | us23 | USA | CT | New Haven | 41°18'26.4"N 72°56'10.7"W | T | 2010 | 6/22/2010 |
| 708 | 120716120S1 | us23 | USA | CT | Salem | 41°29'25.5"N 72°16'48.5"W | T | 2012 | 7/18/2012 |
| 122316 | 120725134S1 | us23.v | USA | CT | Colchester | 41°34'33.5"N 72°20'00.3"W | T | 2012 | 7/26/2012 |
| 122320 | 120815182S1 | us23.v | USA | CT | Coventry | 41°47'05.4"N 72°20'33.5"W | T | 2012 | 8/16/2012 |
| 1112 | na | us23.v | USA | CT | Suffield | 41°59'25.6"N 72°42'15.6"W | T | 2012 | 12/11/2012 |
| 1717 | 140825206S1 | us23 | USA | CT | Kent | 41°43'26.9"N 73°28'42.1"W | T | 2014 | 8/25/2014 |
| 1208 | na | us23 | USA | DE | na | na | na | 2013 | 5/12/2013 |
| 1260 | na | us23 | USA | DE | na | na | na | 2013 | 5/12/2013 |
| 12232 | 120429027S1 | us23 | USA | FL | Jupiter | 26°56'02.6"N 80°05'44.0"W | T | 2012 | 4/28/2012 |
| 13231 | 130104001S1 | us23 | USA | FL | na | na | P | 2013 | 1/4/2013 |
| 1011 | 130115005S1 | us23 | USA | FL | Collier Co | 26°14'52.4"N 81°35'29.2"W | T | 2013 | 1/16/2013 |
| 1040 | 130115004S1 | us23 | USA | FL | Collier Co | 26°14'52.4"N 81°35'29.2"W | P | 2013 | 1/16/2013 |
| 13232 | 130219012S1 | us23 | USA | FL | Palm Beach Co | 26°46'54.3"N 80°21'25.6"W | T | 2013 | 2/6/2013 |
| 1038 | 130410017S1 | us23 | USA | FL | Manatee | 27°31'01.6"N 82°12'23.9"W | T | 2013 | 4/10/2013 |
| 952 | na | us23 | USA | FL | Hendry Co | 26°34'55.5"N 81°09'11.0"W | T | 2014 | na |
| 956 | na | us23 | USA | FL | Naples | 26°08'32.4"N 81°47'45.4"W | T | 2014 | na |
| 957 | na | us23 | USA | FL | Ruskin | 27°43'14.2"N 82°26'03.4"W | T | 2014 | na |
| 958 | na | us23 | USA | FL | Parrish | 27°35'15.8"N 82°25'30.3"W | T | 2014 | na |
| 1537 | na | us23 | USA | FL | Naples | 26°08'32.4"N 81°47'45.4"W | T | 2014 | na |
| 1753 | na | us23 | USA | FL | Naples | 26°08'32.4"N 81°47'45.4"W | T | 2014 | na |
| 1723 | 140916274S1 | us23 | USA | ID | Blackfoot | 43°11'27.0"N 112°20'45.4"W | P | 2014 | 9/16/2014 |
| 132348 | 131119293S1 | us23 | USA | IN | Miami Co | 40°48'27.4"N 86°03'52.2"W | T | 2013 | 11/18/2013 |
| 132342 | na | us23 | USA | MA | Amherst | 42°22'26.2"N 72°31'12.9"W | T | 2013 | 10/8/2013 |
| 132344 | na | us23 | USA | MA | Montague | 42°32'04.2"N 72°32'09.0"W | T | 2013 | 11/4/2013 |
| 1734 | 140729078S1 | us23 | USA | MA | South Deerfield | 42°28'40.1"N 72°36'30.3"W | P | 2014 | 7/29/2014 |
| 1733 | 140813121S1 | us23 | USA | MA | Concord | 42°27'37.4"N 71°20'59.0"W | T | 2014 | 8/13/2014 |
| 1618 | 140822174S1 | us23 | USA | MA | Merrimac | 42°50'01.5"N 71°00'03.8"W | T | 2014 | 8/22/2014 |
| 122323 | 120813177R | us23 | USA | MD | na | na | T | 2012 | 8/21/2012 |
| 820 | na | us23.v | USA | MD | na | na | T | 2012 | 10/16/2012 |
| 132337 | na | us23 | USA | MD | Cumberland | 39°39'10.4"N 78°45'47.4"W | T | 2013 | 9/10/2013 |
| 1742 | 140813117S1 | us23 | USA | MD | Grantsville | 39°41'42.5"N 79°09'05.6"W | T | 2014 | 8/13/2014 |
| 1495 | na | us23 | USA | ME | na | na | T | 2011 | 8/25/2011 |
| 2013 | na | us23 | USA | ME | Bridgewater | 46°25'36.8"N 67°50'50.7"W | P | 2011 | 9/9/2011 |
| 112319 | 110928001 | us23 | USA | ME | Mapleton | 46°40'52.2"N 68°09'32.1"W | P | 2011 | 9/28/2011 |
| 1424 | 120712116S1 | us23 | USA | ME | Fryeburg | 44°01'03.0"N 70°59'03.5"W | P | 2012 | 7/24/2012 |
| 803 | 120721126S1 | us23.v | USA | ME | Exeter | 44°57'44.1"N 69°07'19.5"W | P | 2012 | 7/24/2012 |
| 468 | 120725131S1 | us23 | USA | ME | Littleton | 46°13'51.4"N 67°50'19.3"W | P | 2012 | 7/26/2012 |
| 697 | 120725133S1 | us23 | USA | ME | Fort Kent | 47°15'31.5"N 68°35'08.3"W | P | 2012 | 7/26/2012 |
| 122318 | 120809162S1 | us23 | USA | ME | Troy | 44°40'40.4"N 69°15'27.1"W | T | 2012 | 8/10/2012 |
| 122319 | 120809161S1 | us23.v | USA | ME | Charleston | 45°05'12.2"N 69°02'38.5"W | P | 2012 | 8/10/2012 |
| 122340 | 120814180S1 | us23 | USA | ME | Waldo Co | 44°30'43.6"N 69°10'32.2"W | T | 2012 | 8/15/2012 |
| 122341 | 120814181S1 | us23 | USA | ME | St. David | 47°20'14.5"N 68°13'53.6"W | P | 2012 | 8/15/2012 |
| 122344 | 120814179S1 | us23 | USA | ME | na | na | P | 2012 | 8/15/2012 |
| 646 | 120823216S1 | us23 | USA | ME | Old Town | 44°56'06.7"N 68°39'03.5"W | T | 2012 | 8/24/2012 |
| 579 | na | us23 | USA | ME | na | na | P | 2012 | 11/15/2012 |
| 132313 | 130807102S1 | us23 | USA | ME | Houlton | 46°07'38.1"N 67°50'30.9"W | P | 2013 | 8/8/2013 |
| 132322 | 130827183S1 | us23 | USA | ME | Aroostook Co | 46°54'50.1"N 68°24'14.2"W | P | 2013 | 8/28/2013 |
| 132323 | 130827184S1 | us23 | USA | ME | Aroostook Co | 46°54'50.1"N 68°24'14.2"W | T | 2013 | 8/28/2013 |
| 1630 | 140813123S1 | us23 | USA | ME | Exeter | 44°57'43.2"N 69°07'18.3"W | P | 2014 | 8/13/2014 |
| 1663 | 140814128S1 | us23 | USA | ME | Frenchville | 47°16'41.5"N 68°22'15.0"W | P | 2014 | 8/14/2014 |
| 1664 | 140814127S1 | us23 | USA | ME | Littleton | 46°13'57.4"N 67°50'32.9"W | P | 2014 | 8/14/2014 |
| 1737 | 140814129S1 | us23 | USA | ME | Grand Isle | 47°18'17.9"N 68°09'20.3"W | P | 2014 | 8/14/2014 |
| 1611 | 140822186S1 | us23 | USA | ME | Corinna | 44°55'19.6"N 69°16'03.0"W | P | 2014 | 8/22/2014 |
| 1607 | 140828220S1 | us23 | USA | ME | Winslow | 44°32'47.3"N 69°37'09.2"W | T | 2014 | 8/28/2014 |
| 834 | na | us23 | USA | MN | na | na | P | 2012 | 10/9/2012 |
| 1308S1 | na | us23 | USA | na | na | na | na | 2013 | na |
| 12233 | 120613051S1 | us23 | USA | NC | Camden Co | 36°26'43.5"N 76°16'03.1"W | P | 2012 | 5/30/2012 |
| 12237 | 120608263S1 | us23 | USA | NC | Belvidere | 36°16'10.6"N 76°32'08.9"W | T | 2012 | 6/6/2012 |
| 702 | 120608261S1 | us23.v | USA | NC | Chowan Co | 36°10'19.4"N 76°37'48.2"W | T | 2012 | 6/6/2012 |
| 727 | 120618078S1 | us23 | USA | NC | Pasquotank Co | 36°20'35.7"N 76°21'03.5"W | na | 2012 | 6/19/2012 |
| 13236 | 130722065S1 | us23 | USA | NC | Fleetwood | 36°18'25.9"N 81°30'49.0"W | T | 2013 | 7/25/2013 |
| 1542 | 140718039S1 | us23 | USA | NC | Mills River | 35°23'20.2"N 82°33'54.3"W | T | 2014 | 7/18/2014 |
| 1615 | 140825204S1 | us23 | USA | NC | Mills River | 35°23'20.2"N 82°33'54.3"W | T | 2014 | 8/25/2014 |
| 1726 | 140909262S1 | us23 | USA | NC | Mills River | 35°23'20.2"N 82°33'54.3"W | T | 2014 | 9/9/2014 |
| 1729 | 140909262S1 | us23 | USA | NC | Mills River | 35°23'20.2"N 82°33'54.3"W | T | 2014 | 9/9/2014 |
| 833 | na | us23 | USA | ND | na | na | P | 2012 | 10/9/2012 |
| 694 | 120724129S1 | us23 | USA | NY | Calverton | 40°54'25.5"N 72°44'54.9"W | T | 2012 | 7/25/2012 |
| 10233 | na | us23 | USA | NH | Grafton Co | 44°02'07.2"N 71°58'02.5"W | T | 2010 | 10/6/2010 |
| 1239 | na | us23 | USA | NH | na | na | T | 2011 | 8/26/2011 |
| 1129 | 120911277S1 | us23.v | USA | NJ | Mercer Co | 40°16'55.1"N 74°42'36.2"W | T | 2012 | 8/30/2012 |
| 122329 | na | us23 | USA | NJ | Morris Co | 40°47'48.4"N 74°28'16.0"W | T | 2012 | 9/6/2012 |
| 122330 | na | us23 | USA | NJ | Sussex Co | 41°08'25.2"N 74°40'38.5"W | T | 2012 | 9/7/2012 |
| 1222 | na | us23 | USA | NJ | Great Meadows | 40°52'20.6"N 74°54'43.2"W | T | 2012 | 9/10/2012 |
| 1189 | na | us23 | USA | NY | Suffolk Co | 40°54'21.4"N 72°59'33.6"W | T | 2011 | 6/28/2011 |
| 11231 | na | us23 | USA | NY | Suffolk Co | 40°54'21.4"N 72°59'33.6"W | T | 2011 | 6/28/2011 |
| 11232 | na | us23 | USA | NY | Suffolk Co | 40°54'21.4"N 72°59'33.6"W | P | 2011 | 6/28/2011 |
| 11234 | na | us23 | USA | NY | Suffolk Co | 40°54'21.4"N 72°59'33.6"W | T | 2011 | 6/28/2011 |
| 112323 | na | us23 | USA | NY | Suffolk Co | 40°54'21.4"N 72°59'33.6"W | P | 2011 | 6/28/2011 |
| 11237 | na | us23 | USA | NY | Suffolk Co | 40°54'21.4"N 72°59'33.6"W | T | 2011 | 7/7/2011 |
| 11238 | na | us23 | USA | NY | Suffolk Co | 40°54'21.4"N 72°59'33.6"W | T | 2011 | 7/7/2011 |
| 2027 | na | us23 | USA | NY | Chazy | 44°53'27.5"N 73°26'11.2"W | T | 2011 | 9/7/2011 |
| 1108 | na | us23 | USA | NY | Suffolk Co | 40°54'21.4"N 72°59'33.6"W | T | 2011 | 10/7/2011 |
| 817 | 120529031S1 | us23 | USA | NY | Suffolk Co | 40°54'21.4"N 72°59'33.6"W | P | 2012 | 5/30/2012 |
| 12239 | 120621086S1 | us23 | USA | NY | Suffolk Co | 40°54'21.4"N 72°59'33.6"W | T | 2012 | 6/21/2012 |
| 122312 | 120703106S1 | us23 | USA | NY | Suffolk Co | 40°54'21.4"N 72°59'33.6"W | T | 2012 | 7/2/2012 |
| 122315 | na | us23.v | USA | NY | Suffolk Co | 40°54'21.4"N 72°59'33.6"W | T | 2012 | 7/14/2012 |
| 707 | 120725130S1 | us23.v | USA | NY | Dutchess Co | 41°48'40.9"N 73°43'00.6"W | T | 2012 | 7/20/2012 |
| 122336 | 120822206S1 | us23 | USA | NY | Clinton Co | 44°46'45.7"N 73°36'48.9"W | T | 2012 | 8/2/2012 |
| 122337 | 120802148S1 | us23 | USA | NY | Washington Co | 43°18'04.2"N 73°24'22.5"W | T | 2012 | 8/3/2012 |
| 122317 | 120803149S1 | us23 | USA | NY | Wyoming Co | 42°42'34.4"N 78°14'26.8"W | P | 2012 | 8/8/2012 |
| 122338 | 120806155S1 | us23 | USA | NY | Tompkins Co | 42°26'12.3"N 76°32'24.0"W | T | 2012 | 8/9/2012 |
| 122342 | 120815185S1 | us23 | USA | NY | Suffolk Co | 40°54'43.8"N 72°42'45.1"W | T | 2012 | 8/15/2012 |
| 122343 | 120815184S1 | us23 | USA | NY | Suffolk Co | 40°54'43.8"N 72°42'45.1"W | T | 2012 | 8/15/2012 |
| 122322 | 120822204S1 | us23 | USA | NY | Columbia Co | 42°17'03.5"N 73°38'08.6"W | T | 2012 | 8/17/2012 |
| 811 | 120821199S1 | us23.v | USA | NY | Varna | 42°27'20.9"N 76°26'17.5"W | T | 2012 | 8/20/2012 |
| 532 | 1208212000S1 | us23.v | USA | NY | Fulton Co | 43°05'32.9"N 74°21'22.5"W | T | 2012 | 8/21/2012 |
| 807 | 120821197S1 | us23 | USA | NY | Bemus Pt | 42°09'49.4"N 79°23'16.6"W | T | 2012 | 8/22/2012 |
| 812 | 120821198S1 | us23.v | USA | NY | Whitesboro | 43°07'19.0"N 75°17'32.1"W | T | 2012 | 8/22/2012 |
| 813 | 120822205S1 | us23.v | USA | NY | Salem | 43°10'15.5"N 73°19'30.3"W | T | 2012 | 8/23/2012 |
| 809 | 120823213S1 | us23 | USA | NY | New Paltz | 41°44'51.6"N 74°05'07.1"W | T | 2012 | 8/24/2012 |
| 122324 | 120911273S1 | us23 | USA | NY | Saranac Lake | 44°19'46.6"N 74°08'11.2"W | T | 2012 | 8/24/2012 |
| 778 | 120830242S1 | us23.v | USA | NY | Baldwinsville | 43°09'32.1"N 76°20'07.1"W | T | 2012 | 8/31/2012 |
| 122328 | 120905260S1 | us23 | USA | NY | Wayne Co | 43°11'09.0"N 77°07'07.9"W | T | 2012 | 9/5/2012 |
| 773 | na | us23.v | USA | NY | Chenango Co | 42°29'06.1"N 75°34'34.5"W | T | 2012 | 9/14/2012 |
| 1020 | na | us23.v | USA | NY | Columbia Co | 42°17'03.5"N 73°38'08.6"W | T | 2012 | 9/17/2012 |
| 776 | 120917282S1 | us23.v | USA | NY | Canandaigua |  | T | 2012 | 9/18/2012 |
| 558 | na | us23 | USA | NY | Erie Co | 42°42'37.4"N 78°39'46.2"W | T | 2012 | 10/12/2012 |
| 769 | na | us23.v | USA | NY | Canton | 44°35'44.3"N 75°10'13.4"W | T/P | 2012 | 10/17/2012 |
| 136140 | na | us23 | USA | NY | Geneva | 42°52'03.1"N 76°59'12.4"W | T | 2013 | 8/19/2013 |
| 132318 | na | us23 | USA | NY | Stuyvesant | 42°23'28.6"N 73°46'53.6"W | T | 2013 | 8/22/2013 |
| 132319 | na | us23 | USA | NY | Salem | 43°10'26.9"N 73°19'39.8"W | T | 2013 | 8/22/2013 |
| 132320 | 130820157S1 | us23 | USA | NY | Avoca | 42°24'33.8"N 77°25'14.8"W | T | 2013 | 8/22/2013 |
| 132336 | 130902210S1 | us23 | USA | NY | Newark | 43°02'47.4"N 77°05'43.6"W | P | 2013 | 9/9/2013 |
| 132340 | 131002280S1 | us23 | USA | NY | Wilseyville | 42°17'26.9"N 76°22'41.6"W | T | 2013 | 10/3/2013 |
| 1703 | 140718040S1 | us23 | USA | NY | Columbia Co | 42°17'03.5"N 73°38'08.6"W | P | 2014 | 7/18/2014 |
| 1627 | 140804089S1 | us23 | USA | NY | Penn Yan | 42°39'38.2"N 77°03'18.0"W | T | 2014 | 8/4/2014 |
| 1662 | 140814124S1 | us23 | USA | NY | Canandaigua | 42°53'13.5"N 77°17'01.8"W | T | 2014 | 8/14/2014 |
| 1638 | 140820155S1 | us23 | USA | NY | Batavia | 42°59'52.6"N 78°11'15.8"W | T | 2014 | 8/20/2014 |
| 1609 | 140827215S1 | us23 | USA | NY | Oneida Co | 43°12'57.2"N 75°28'11.5"W | T | 2014 | 8/27/2014 |
| 1610 | 140827214S1 | us23 | USA | NY | Seneca Co | 42°50'35.1"N 76°49'30.8"W | T | 2014 | 8/27/2014 |
| 1714 | 140902236S1 | us23 | USA | NY | St. Lawrence Co | 44°31'33.2"N 75°09'07.5"W | P | 2014 | 9/2/2014 |
| 1697 | 140922278S1 | us23 | USA | NY | Marcellus | 42°58'58.2"N 76°20'21.0"W | T | 2014 | 9/22/2014 |
| 1858 | na | us23 | USA | NY | Trumansburg | 42°32'33.1"N 76°40'04.8"W | T | 2014 | na |
| 990 | 120822207S1 | us23.v | USA | OH | Pike | 39°04'51.7"N 83°05'48.4"W | T | 2012 | 8/14/2012 |
| 132314 | 130809107S1 | us23 | USA | OH | Wooster | 40°48'15.4"N 81°56'01.3"W | T | 2013 | 8/13/2013 |
| 132325 | na | us23 | USA | OH | Summit Co | 41°10'15.1"N 81°31'00.2"W | T | 2013 | 8/29/2013 |
| 132327 | na | us23 | USA | OH | Massillon | 40°47'46.8"N 81°31'19.1"W | T | 2013 | 8/29/2013 |
| 9231 | na | us23 | USA | PA | Blair Co | 40°33'17.5"N 78°24'49.1"W | P | 2009 | na |
| 2055 | 110707001 | us23 | USA | PA | Loretto | 40°30'10.4"N 78°37'50.9"W | P | 2011 | 7/7/2011 |
| 2057 | 110922001 | us23 | USA | PA | na | na | P | 2011 | 9/22/2011 |
| 112317 | 110927001 | us23 | USA | PA | Lehigh Co | 40°36'50.6"N 75°35'46.5"W | T | 2011 | 9/27/2011 |
| 628 | 111006006 | us23 | USA | PA | Centre Co | 40°52'01.3"N 77°35'04.6"W | T | 2011 | 10/6/2011 |
| 112322 | 111018001 | us23 | USA | PA | Bedford | 40°01'07.1"N 78°30'14.3"W | T | 2011 | 10/18/2011 |
| 795 | 120607039S1 | us23 | USA | PA | Mifflin Co | 40°36'16.0"N 77°38'56.7"W | T | 2012 | 6/8/2012 |
| 12238 | 120613059S1 | us23.v | USA | PA | Schuylkill Co | 40°42'12.6"N 76°12'37.7"W | P | 2012 | 6/14/2012 |
| 805 | 120614065S1 | us23 | USA | PA | Mifflin Co | 40°36'16.0"N 77°38'56.7"W | T | 2012 | 6/15/2012 |
| 506 | 120618075S1 | us23 | USA | PA | Chester Co | 39°58'46.7"N 75°44'36.2"W | P | 2012 | 6/19/2012 |
| 644 | na | us23.v | USA | PA | na | na | T | 2012 | 7/31/2012 |
| 122335 | 120731146S1 | us23 | USA | PA | Lehigh Co | 40°36'50.6"N 75°35'46.5"W | T | 2012 | 8/2/2012 |
| 1153 | 120809159S1 | us23 | USA | PA | Cumberland Co | 40°09'13.6"N 77°13'56.1"W | T | 2012 | 8/10/2012 |
| 645 | 120813173S1 | us23 | USA | PA | Pennsylvania Furnace | 40°42'23.3"N 78°00'13.5"W | T | 2012 | 8/14/2012 |
| 122345 | 120816187S1 | us23 | USA | PA | Pennsylvania Furnace | 40°42'23.3"N 78°00'13.5"W | P | 2012 | 8/17/2012 |
| 122326 | 120827228S1 | us23 | USA | PA | Northumberland Co | 40°54'13.8"N 76°41'22.0"W | T | 2012 | 8/28/2012 |
| 673 | 120827227S1 | us23.v | USA | PA | Cambria Co | 40°31'48.3"N 78°41'08.8"W | T | 2012 | 8/28/2012 |
| 520 | 120904255S1 | us23 | USA | PA | Indiana Co | 40°40'52.9"N 79°04'23.4"W | T | 2012 | 9/5/2012 |
| 678 | 120904256S1 | us23.v | USA | PA | Montour Co | 41°02'04.0"N 76°39'32.0"W | T | 2012 | 9/5/2012 |
| 13239 | 130731080S1 | us23 | USA | PA | Alleghany Co | 40°27'18.0"N 80°05'33.9"W | T | 2013 | 8/6/2013 |
| 132317 | 130820159S1 | us23 | USA | PA | Manheim | 40°09'48.2"N 76°23'36.0"W | T | 2013 | 8/22/2013 |
| 1637 | 140630023S1 | us23 | USA | PA | Lancaster Co | 40°03'32.7"N 76°13'43.3"W | P | 2014 | 6/30/2014 |
| 1636 | 140721050S1 | us23 | USA | PA | Centre Co | 40°49'24.2"N 77°44'57.9"W | T | 2014 | 7/21/2014 |
| 1600 | 140806096S1 | us23 | USA | PA | Lehigh Co | 40°36'50.6"N 75°35'46.5"W | T | 2014 | 8/6/2014 |
| 1666 | 140815136S1 | us23 | USA | PA | Schuylkill Co | 40°42'12.6"N 76°12'37.7"W | T | 2014 | 8/15/2014 |
| 1671 | 140815134S1 | us23 | USA | PA | Indiana Co | 40°40'52.9"N 79°04'23.4"W | T | 2014 | 8/15/2014 |
| 1727 | 140828218S1 | us23 | USA | PA | Chambersburg | 39°56'15.2"N 77°39'45.0"W | T | 2014 | 8/28/2014 |
| 1602 | 140903242S1 | us23 | USA | PA | Westmoreland Co | 40°20'23.4"N 79°27'37.6"W | T | 2014 | 9/3/2014 |
| 112311 | 110819001 | us23 | USA | RI | na | na | T | 2011 | 8/19/2011 |
| 132312 | 130806094S1 | us23 | USA | RI | Cranston | 41°46'50.0"N 71°26'49.7"W | T | 2013 | 8/7/2013 |
| 112310 | na | us23 | USA | VA | Greene Co | 38°17'46.0"N 78°28'48.2"W | P | 2011 | 7/12/2011 |
| 1757 | 140812111S1 | us23 | USA | VA | Washington | 38°42'45.9"N 78°09'38.5"W | T | 2014 | 8/14/2014 |
| 1608 | 140820160S1 | us23 | USA | VA | Blacksburg | 37°13'43.6"N 80°25'01.6"W | T | 2014 | 8/20/2014 |
| 112312 | na | us23.v | USA | WI | Vernon Co | 43°36'40.5"N 90°51'14.3"W | P | 2009 | 8/23/2011 |
| 10232 | na | us23 | USA | WI | Waukesha Co | 43°00'26.3"N 88°15'32.5"W | T | 2010 | 8/4/2010 |
| 1237 | na | us23 | USA | WI | na | na | T | 2011 | 8/23/2011 |
| 112313 | na | us23 | USA | WI | Waukesha Co | 43°00'26.3"N 88°15'32.5"W | T | 2011 | 8/23/2011 |
| 767 | na | us23.v | USA | WI | Barron Co | 45°26'42.8"N 91°54'26.1"W | na | 2012 | 10/18/2012 |
| 132330 | na | us23 | USA | WI | na | na | na | 2013 | 9/1/2013 |
| 1247 | na | us24 | USA | ME | Caribou | 46°51'46.2"N 67°59'59.7"W | P | 2011 | 7/21/2011 |
| 1426 | na | us24 | USA | ME | na | na | P | 2011 | 8/25/2011 |
| 1268 | na | us24 | USA | MN | Freeborn Co | 43°41'39.9"N 93°18'07.8"W | P | 2011 | 7/19/2011 |
| 1409 | na | us24 | USA | MN | Sabin | 46°46'46.4"N 96°39'16.1"W | na | 2011 | 11/17/2011 |
| 1344 | na | us24 | USA | MT | na | na | P | 2010 | 10/15/2010 |
| 1345 | na | us24 | USA | MT | na | na | P | 2010 | 10/15/2010 |
| 1346 | na | us24 | USA | MT | na | na | P | 2010 | 10/15/2010 |
| 1348 | na | us24 | USA | MT | na | na | P | 2010 | 10/15/2010 |
| 700 | 120613060S1 | us24 | USA | NC | Camden Co | 36°26'43.5"N 76°16'03.1"W | P | 2012 | 6/6/2012 |
| ND884_5 | na | us24 | USA | ND | Grand Forks | 47°55'32.6"N 97°02'37.7"W | P | 2009 | na |
| ND888 | na | us24 | USA | ND | Grand Forks | 47°55'32.6"N 97°02'37.7"W | P | 2009 | na |
| ND889_1 | na | us24 | USA | ND | Grand Forks | 47°55'32.6"N 97°02'37.7"W | P | 2009 | na |
| 1198 | na | us24 | USA | ND | na | na | P | 2011 | 8/23/2011 |
| 1246 | na | us24 | USA | ND | na | na | P | 2011 | 8/23/2011 |
| 1249 | na | us24 | USA | ND | na | na | P | 2011 | 8/23/2011 |
| 2041 | na | us24 | USA | ND | na | na | P | 2011 | 8/23/2011 |
| 1219 | na | us24 | USA | ND | Hoople |  | na | 2011 | 11/17/2011 |
| 1513 | na | us24 | USA | ND | na | na | na | 2011 | 11/17/2011 |
| 2008 | na | us24 | USA | ND | Grafton | 48°24'42.5"N 97°24'36.7"W | na | 2011 | 11/17/2011 |
| US110157 | na | us24 | USA | ND | Grand Forks | 47°55'32.6"N 97°02'37.7"W | na | 2011 | 11/17/2011 |
| 1326 | na | us24 | USA | ND | na | na | P | 2012 | 6/5/2012 |
| 630 | na | us24 | USA | NY | Freeville | 42°30'50.9"N 76°20'50.3"W | P | 2011 | 10/12/2011 |
| 685 | na | us24 | USA | NY | Freeville | 42°30'50.9"N 76°20'50.3"W | P | 2011 | 10/12/2011 |
| 1228 | na | us24 | USA | NY | Freeville | 42°30'50.9"N 76°20'50.3"W | P | 2011 | 10/12/2011 |
| 1273 | na | us24 | USA | NY | Freeville | 42°30'50.9"N 76°20'50.3"W | P | 2011 | 10/12/2011 |
| 1250 | 110830001 | us24 | USA | OR | Corvallis | 44°33'54.5"N 123°16'01.4"W | Y | 2011 | 8/30/2011 |
| 1322 | 130806099S1 | us24 | USA | OR | Lebanon | 44°32'12.7"N 122°54'18.3"W | P | 2013 | 8/7/2013 |
| 1476 | 130905227S1 | us24 | USA | OR | Lebanon | 44°32'12.7"N 122°54'18.3"W | T | 2013 | 9/6/2013 |
| 1324 | 130923275S1 | us24 | USA | OR | Corvallis | 44°33'54.5"N 123°16'01.4"W | T | 2013 | 9/25/2013 |
| 13111 | 130923276S1 | us24 | USA | OR | Corvallis | 44°33'54.5"N 123°16'01.4"W | P | 2013 | 9/25/2013 |
| 1628 | na | us24 | USA | OR | Philomath | 44°32'24.7"N 123°22'10.9"W | P | 2014 | na |
| 1634 | na | us24 | USA | OR | Lebenon | 44°32'12.7"N 122°54'18.3"W | P | 2014 | na |
| 1635 | na | us24 | USA | OR | Corvallis | 44°33'54.5"N 123°16'01.4"W | T | 2014 | na |
| 1312 | na | us24 | USA | WA | na | na | P | 2011 | 10/6/2011 |
| 1335 | na | us24 | USA | WA | na | na | P | 2011 | 10/6/2011 |
| 1339 | na | us24 | USA | WA | na | na | P | 2011 | 10/6/2011 |

^x^ Isolates submitted prior to the USAblight project initiation in 2011 do not have USAblight IDs (http://www.usablight.org). Several isolates submitted during and after 2011 were not submitted to the USAblight database, and therefore also do not have USAblight IDs. na = not available.

^y^ Clonal lineages with a variant SSR genotype are denoted by (.v).

^z^ Isolate geographic coordinates are approximate.
