# Supplementary material for: Genetic Variation within Clonal Lineages of Phytophthora infestans Revealed through Genotyping-By-Sequencing, and Implications for Late Blight Epidemiology
Source: PLoS One. 2016 Nov 3;11(11):e0165690. doi: 10.1371/journal.pone.0165690 (PMC5094694; doi:10.1371/journal.pone.0165690)
Supplement: S2 Table — (DOCX) [file pone.0165690.s005.docx]

**S2 Table.** Number of *P. infestans* isolates within each lineage organized by year and collection location.

| **Location^y^** | **1994** | **2004** | **2005** | **2008** | **2009** | **2010** | **2011** | **2012** | **2013** | **2014** | **2015** | **Sum^z^** |
| --- | --- | --- | --- | --- | --- | --- | --- | --- | --- | --- | --- | --- |
|  |  | **US-8** | | | | | | | | | | |
| **ID** | 0 | 0 | 0 | 0 | 0 | 0 | 1 | 0 | 0 | 0 | 0 | **1** |
| **MA** | 0 | 0 | 0 | 0 | 0 | 0 | 0 | 0 | 1 | 0 | 0 | **1** |
| **ME** | 0 | 0 | 0 | 1 | 0 | 0 | 0 | 0 | 0 | 0 | 0 | **1** |
| **NY** | 1 | 1 | 0 | 1 | 3 | 2 | 0 | 0 | 1 | 0 | 0 | **9** |
| **OR** | 0 | 0 | 0 | 0 | 0 | 0 | 1 | 0 | 0 | 0 | 0 | **1** |
| **PA** | 0 | 0 | 0 | 0 | 1 | 1 | 1 | 0 | 0 | 0 | 0 | **3** |
| **VA** | 0 | 0 | 0 | 0 | 0 | 0 | 0 | 1 | 0 | 0 | 0 | **1** |
| **WA** | 0 | 0 | 0 | 0 | 0 | 0 | 3 | 0 | 1 | 1 | 0 | **5** |
| **On, Can** | 0 | 0 | 0 | 0 | 0 | 6 | 0 | 0 | 0 | 0 | 0 | **6** |
| **Sum** | **1** | **1** | **0** | **2** | **4** | **9** | **6** | **1** | **3** | **1** | **0** | **28** |
|  |  | **US-11** | | | | | | | | | | |
| **CA** | 0 | 0 | 0 | 0 | 0 | 0 | 3 | 2 | 1 | 0 | 1 | **7** |
| **FL** | 0 | 0 | 0 | 0 | 0 | 0 | 0 | 2 | 0 | 0 | 0 | **2** |
| **NC** | 0 | 0 | 0 | 0 | 0 | 0 | 0 | 1 | 0 | 0 | 0 | **1** |
| **NY** | 0 | 0 | 1 | 0 | 0 | 0 | 1 | 0 | 0 | 0 | 0 | **2** |
| **OR** | 0 | 0 | 1 | 0 | 0 | 0 | 6 | 0 | 1 | 0 | 0 | **8** |
| **WA** | 0 | 0 | 0 | 0 | 0 | 0 | 0 | 7 | 0 | 0 | 0 | **7** |
| **Sum** | **0** | **0** | **2** | **0** | **0** | **0** | **10** | **12** | **2** | **0** | **1** | **27** |
|  |  | **US-23** | | | | | | | | | | |
| **CT** | 0 | 0 | 0 | 0 | 0 | 1 | 0 | 4 | 0 | 1 | 0 | **6** |
| **DE** | 0 | 0 | 0 | 0 | 0 | 0 | 0 | 0 | 2 | 0 | 0 | **2** |
| **FL** | 0 | 0 | 0 | 0 | 0 | 0 | 0 | 1 | 5 | 6 | 0 | **12** |
| **ID** | 0 | 0 | 0 | 0 | 0 | 0 | 0 | 0 | 0 | 1 | 0 | **1** |
| **IN** | 0 | 0 | 0 | 0 | 0 | 0 | 0 | 0 | 1 | 0 | 0 | **1** |
| **MA** | 0 | 0 | 0 | 0 | 0 | 0 | 0 | 0 | 2 | 3 | 0 | **5** |
| **MD** | 0 | 0 | 0 | 0 | 0 | 0 | 0 | 2 | 1 | 1 | 0 | **4** |
| **ME** | 0 | 0 | 0 | 0 | 0 | 0 | 3 | 11 | 3 | 6 | 0 | **23** |
| **MN** | 0 | 0 | 0 | 0 | 0 | 0 | 0 | 1 | 0 | 0 | 0 | **1** |
| **NC** | 0 | 0 | 0 | 0 | 0 | 0 | 0 | 4 | 1 | 4 | 0 | **9** |
| **ND** | 0 | 0 | 0 | 0 | 0 | 0 | 0 | 1 | 0 | 0 | 0 | **1** |
| **NH** | 0 | 0 | 0 | 0 | 0 | 1 | 1 | 0 | 0 | 0 | 0 | **2** |
| **NJ** | 0 | 0 | 0 | 0 | 0 | 0 | 0 | 4 | 0 | 0 | 0 | **4** |
| **NY** | 0 | 0 | 0 | 0 | 0 | 0 | 9 | 27 | 6 | 9 | 0 | **51** |
| **OH** | 0 | 0 | 0 | 0 | 0 | 0 | 0 | 1 | 3 | 0 | 0 | **4** |
| **PA** | 0 | 0 | 0 | 0 | 1 | 0 | 5 | 13 | 2 | 7 | 0 | **28** |
| **RI** | 0 | 0 | 0 | 0 | 0 | 0 | 1 | 0 | 1 | 0 | 0 | **2** |
| **VA** | 0 | 0 | 0 | 0 | 0 | 0 | 1 | 0 | 0 | 2 | 0 | **3** |
| **WI** | 0 | 0 | 0 | 0 | 1 | 1 | 2 | 1 | 1 | 0 | 0 | **6** |
| **na** | 0 | 0 | 0 | 0 | 0 | 0 | 0 | 0 | 1 | 0 | 0 | **1** |
| **Sum** | **0** | **0** | **0** | **0** | **2** | **3** | **22** | **70** | **29** | **40** | **0** | **166** |
|  |  | **US-24** | | | | | | | | | | |
| **ME** | 0 | 0 | 0 | 0 | 0 | 0 | 2 | 0 | 0 | 0 | 0 | **2** |
| **MN** | 0 | 0 | 0 | 0 | 0 | 0 | 2 | 0 | 0 | 0 | 0 | **2** |
| **MT** | 0 | 0 | 0 | 0 | 0 | 4 | 0 | 0 | 0 | 0 | 0 | **4** |
| **NC** | 0 | 0 | 0 | 0 | 0 | 0 | 0 | 1 | 0 | 0 | 0 | **1** |
| **ND** | 0 | 0 | 0 | 0 | 3 | 0 | 8 | 1 | 0 | 0 | 0 | **12** |
| **NY** | 0 | 0 | 0 | 0 | 0 | 0 | 4 | 0 | 0 | 0 | 0 | **4** |
| **OR** | 0 | 0 | 0 | 0 | 0 | 0 | 1 | 0 | 4 | 3 | 0 | **8** |
| **WA** | 0 | 0 | 0 | 0 | 0 | 0 | 3 | 0 | 0 | 0 | 0 | **3** |
| **Sum** | **0** | **0** | **0** | **0** | **3** | **4** | **20** | **2** | **4** | **3** | **0** | **36** |

^y^All locations are within the United States except six US-8 isolates collected in Ontario, Canada in 2011.

^z^ One individual from each of the replicated isolates was included in the final isolate totals.
