# Supplementary material for: Genetic Variation within Clonal Lineages of Phytophthora infestans Revealed through Genotyping-By-Sequencing, and Implications for Late Blight Epidemiology
Source: PLoS One. 2016 Nov 3;11(11):e0165690. doi: 10.1371/journal.pone.0165690 (PMC5094694; doi:10.1371/journal.pone.0165690)
Supplement: S3 Table — (DOCX) [file pone.0165690.s006.docx]

**S3 Table.** Allele sizes for eleven SSR markers used to assign isolates to clonal lineages.

|  | **Microsatellite allele sizes (bp)^t^** | | | | | | | | | | |
| --- | --- | --- | --- | --- | --- | --- | --- | --- | --- | --- | --- |
| ***P. infestans* lineage** | **Pi02** | **Pi89** | **4B** | **G11** | **Pi04** | **Pi56** | **Pi63** | **Pi70** | **Pi16** | **Pi33** | **D13** |
| **US-8** | -/163/165 | 179/179 | 213/226 | -/155/155 | 166/170 | 257/257 | 280/280 | 190/190 | 173/177 | 202/202 | 106/110 |
| **US-8.V1^u^** | -/163/165 | **177**/179 | 213/226 | -/155/155 | 166/170 | 257/257 | 280/280 | 190/190 | 173/177 | 202/202 | 106/110 |
| **US-8.V2^v^ isolate 1185** | **160**/163/165 | 179/**190** | 213/226 | -/155/155 | 166/170 | 257/257 | 280/280 | 190/190 | 173/177 | 202/202 | 106/110 |
|  |  |  |  |  |  |  |  |  |  |  |  |
| **US-11** | 154/163/165 | 177/179 | 213/213 | -/131/155 | 166/170 | 255/257 | 280/280 | 190/190 | 177/177 | 202/202 | 108/108 |
| **US-11.V^w^ isolate 815** | 154/**162**/- | 177/**177** | 213/213 | -/131/155 | 166/170 | 255/257 | 280/280 | 190/190 | 177/177 | 202/202 | 108/108 |
|  |  |  |  |  |  |  |  |  |  |  |  |
| **US-23** | 162/164/166 | 179/179 | 213/217 | 140/155/205 | 170/170 | 255/257 | 270/279 | 190/190 | 177/177 | 202/205 | 134/134 |
| **US-23.V1^x^** | 162/164/166 | 179/179 | 213/217 | 140/155/205 | 170/170 | 255/257 | 270/279 | 190/190 | 177/177 | 202/205 | **139**/**139** |
| **US-23.V2^y^ isolate 112312** | 162/164/166 | 179/179 | 213/217 | **-**/155/**155** | 170/170 | 255/257 | 270/279 | 190/190 | 177/177 | 202/205 | **210**/**210** |
| **US-23.V3^z^ isolate 122320** | 162/164/166 | 179/179 | 213/217 | 140/155/205 | 170/170 | 255/257 | 270/279 | 190/190 | 177/177 | 202/205 | **132**/**132** |
|  |  |  |  |  |  |  |  |  |  |  |  |
| **US-24** | -/163/165 | 177/179 | 217/226 | -/155/155 | 166/170 | 257/257 | 280/280 | 190/193 | 173/177 | 202/205 | 106/110 |

^t^ Microsatellite markers used to assign each isolate to a clonal lineage were developed by Lees et al. (2006). Variant alleles are indicated by bold text.

^u^ US-8 isolates 1184 and 2039 varied at marker Pi89.

^v^ US-8 isolate 1185 varied at markers Pi02 and Pi89.

^w^ US-11 isolate 815 varied at markers Pi02 and Pi89.

^x^ Twenty-four US-23 isolates shared the same variant alleles at marker D13, denoted by US-23.v in S1 Table.

^y^ US-23 isolate 112312 varied at markers G11 and D13.

^z^ US-23 isolate 122320 varied at marker D13.
